# New tricyclic systems as photosensitizers towards triple negative breast cancer cells

**DOI:** 10.1007/s12272-022-01414-1

**Published:** 2022-11-18

**Authors:** Marilia Barreca, Angela Maria Ingarra, Maria Valeria Raimondi, Virginia Spanò, Antonio Palumbo Piccionello, Michele De Franco, Luca Menilli, Valentina Gandin, Giorgia Miolo, Paola Barraja, Alessandra Montalbano

**Affiliations:** 1 Department of Biological, Chemical, and Pharmaceutical Sciences and Technologies (STEBICEF), University of Palermo, Via Archirafi 32, 90123 Palermo, Italy; 2 Department of Pharmaceutical and Pharmacological Sciences, University of Padova, Via Marzolo 5, 35131 Padua, Italy

**Keywords:** Pyrrolo[1,2-*h*][1,7]naphthyridinone, Pyrido[2,3-*c*]pyrrolo[1,2-*a*]azepinone, Photosensitizing agents, Phototoxic activity, Triple-negative breast cancer, MDA-MB-231

## Abstract

Nineteen pyrrolo[1,2-*h*][1,7]naphthyridinones and pyrido[2,3-*c*]pyrrolo[1,2-*a*]azepinones were synthesized as new tricyclic systems in which the pyridine ring is annelated to the 6,7-dihydroindolizin-8(5*H*)-one and 5,6,7,8-tetrahydro-9*H*-pyrrole[1,2-*a*]azepine-9-one moieties to obtain potential photosensitizing agents. They were tested for their photoantiproliferative activity on a triple-negative breast cancer cell line, MDA-MB-231, in the dark and under UVA light (2.0 J/cm^2^). We demonstrated that their toxicity, only when exposed to light, was primarily due to the generation of reactive oxygen species while their photodegradation products were not responsible for their activity. The most active compounds exhibited photocytotoxicity with IC_50_ values at low micromolar level inducing a decrease in the intracellular content of thiol, thus triggering cancer cell death through apoptosis. All the pyridone derivatives revealed to be pure photosensitizers with preferential photocytotoxic activity towards cancerous over healthy cells. Altogether, the results obtained confirm pyrrolo[1,2-*h*][1,7]naphthyridinones and pyrido[2,3-*c*]pyrrolo[1,2-*a*]azepinones as promising photosensitisers against triple-negative breast cancer.

**Supplementary Information:**

The online version contains supplementary material available at 10.1007/s12272-022-01414-1.

## Introduction

**Fig. 1:**
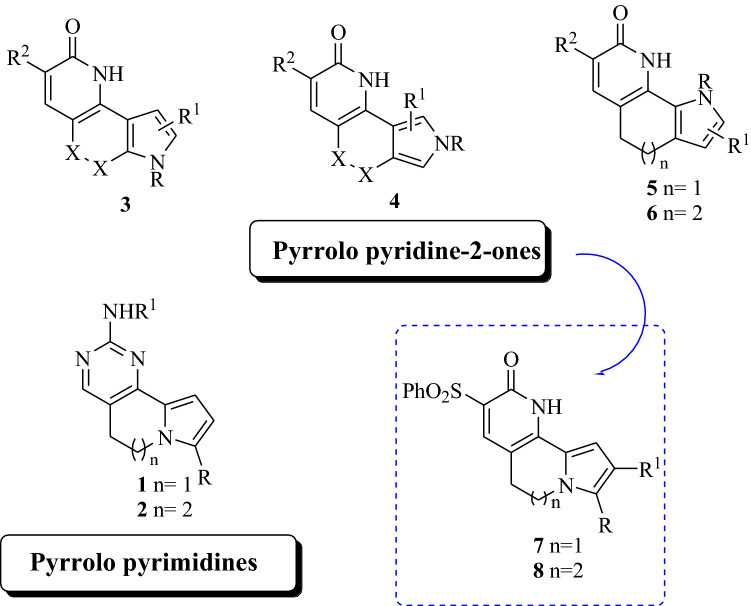
Previously synthetized pyrrolo pyrimido (**1, 2**) and pyrrolo pyridine-2-one systems (**3–6**), and new pyridine scaffolds: pyrrolo[1,2-*h*][1,7]naphthyridinones (**7**) and pyrido[2,3-*c*]pyrrolo[1,2-*a*]azepinones (**8**)

Cancer represents one of the most challenging tasks in drug discovery, and despite several progress have been done, many efforts are still needed. Among the different kind of applications in the treatment of cancer and benign tumours, the use of light is considered a promising non-invasive modality with high selectivity towards malignant target versus normal cells. It can be selectively applied onto a region by illuminating the specific lesion using light with a proper wavelength to activate a tumour-targeted photosensitizer (PS). The latter, by in situ generation of highly cytotoxic reactive oxygen species (ROS), will be able to selectively destroy tumour tissue offering much lower toxic side effects. Over the years there has been a wide number of studies investigating different types of PSs, but the only approved one for clinical use have a tetrapyrrole backbone, such as porphyrins and their analogues. However, their large size is often related to some challenges such as limited tumour specificity and in some cases very important photosensitivity reactions caused by PS accumulation in off-target tissues. Small molecules show several advantages as they typically possess fast distribution throughout the body, rapid clearance, and target accumulation at the tumour site. During our studies aiming at the development of new bioactive heterocyclic compounds (Spanò et al. 2021a, b; Barreca et al. 2022b; Cilibrasi et al. 2022; Labbozzetta et al. 2022), we explored several classes of PSs, and some of them showed high potency, with outstanding selectivity index versus tumour cells inducing mitochondrial apoptotic death (Spanò et al. 2017). We recently reported our studies on two classes of small heterocyclic compounds, namely pyrimido[5,4-*g*]indolizines **1** and pyrimido[4,5-*c*]pyrrolo[1,2-*a*]azepines **2** (Fig. 1) and their antiproliferative activity against the highly refractory triple negative human breast cancer (MDA-MB-231) and vesical human tumour (T24) cell lines, in the dark and under UVA light irradiation. Results indicated very promising antitumour effect, with IC_50_ values up to nanomolar level and strong induction of reactive oxygen species (ROS) and a thiol redox stress responsible of apoptotic cell death (Barreca et al. 2022a). However, some compounds belonging to these series showed activity already in the dark, which is considered not optimal for their use as PSs. In order to extend our insight around their chemical space and inspired by our previous studies indicating the pyridine-2-one moiety as a valuable structural feature within our photoactivable compounds (Barraja et al. 2006, 2010, 2011; Spanò et al. 2017), we decided to explore the effect of the replacement of the pyrimidine moiety generating new classes of tricyclic compounds containing the indolizine and azepine structures incorporating the pyridine-2-one moiety. The choice was supported by our previous evidences related to pyrroloquinolinones **3** and their positional isomers **4, 5** (Fig. 1) which were able to produce phototoxicity in the submicromolar–micromolar range (IC_50_ 0.4–17.5 μM, 0.2–17.5 μM and 0.5–9.3 μM, respectively), high ROS level with the involvement of mitochondria and lysosomes (Barraja et al. 2006, 2010, 2011; Spanò et al. 2017) without generating DNA strand breaks and DNA oxidative damages which represents a great disadvantage in this kind of applications. The enlargement of the central ring of the latter class of derivatives led to pyrrolo[3’,2’:6,7]cyclohepta[1,2-*b*]pyridines **6** (Fig. 1), which maintained phototoxic activity at submicromolar–micromolar level (EC_50_ 0.25–10.70 μM). It was demonstrated that they were able to trigger a rapid apoptotic process through the induction of ROS production with a clear involvement of mitochondrial disfunction and lysosomes.

In this paper we wish to report the synthesis and the evaluation of the photoantiproliferative activity of the new tricyclic systems pyrrolo[1,2-*h*][1,7]naphthyridinones **7** and pyrido[2,3-*c*]pyrrolo[1,2-*a*]azepinones **8** (Fig. 1) and their evaluation as PSs against a very difficult type of breast cancer (BC) to be treated, the triple-negative breast cancer (TNBC).

BC can be described as an etiologically and clinically heterogeneous group of tumours originating from mammary epithelial cells (Lüönd et al. 2021). They can be divided into five molecular subtypes: luminal A, luminal B, HER2-enriched, basal-like (also known as triple-negative breast cancer) and normal-like breast cancer (Ali et al. 2020). These subtypes show differences in incidence, age at diagnosis, prognosis, therapeutic response, and disease progression (Liu et al. 2018). TNBC is an invasive breast carcinoma and represents around 15–20% of newly diagnosed breast cancer (Criscitiello et al. 2012). TNBC is associated with young age at diagnosis (< 40 years) and early metastases despite optimal adjuvant treatment. The metastatic behaviour is predominant in the lungs, central nervous system, and liver (Bergin and Loi 2019). Significant heterogeneity exists within the TNBC class, contrary to the basal-like subtype cancers that present a similar gene expression (Yao et al. 2017). Despite differences within this group, all TNBC subtypes are characterized by the absence of oestrogen receptor (ER), progesterone receptor (PR) and human epidermal growth factor receptor 2 (HER2) expression (Sporikova et al. 2018). Therefore, among all BC subtypes, TNBC has the most limited therapeutic options (Tong et al. 2018) as patients with TNBC do not benefit from therapies that are designed to target the above biomarkers (Tong et al. 2018). All the new synthesized derivatives were tested on TNBC cells, namely MDA-MB-231 human cancer cells, in the dark and upon UVA light. Furthermore, in order to assess their selectivity against tumour cells, all derivatives were additionally screened on a non-transformed human cell line (HEK293 cells). Finally, selected mechanistic investigations were also performed with the aim to deeply characterize their mechanism of action and to evaluate the cell death pathway triggered by the most representative compounds.

## Materials and methods

### Chemistry

All melting points were taken on a Büchi melting point M-560 apparatus. IR spectra were determined in bromoform with a Shimadzu FT/IR 8400S spectrophotometer. ^1^H and ^13^C NMR spectra were measured at 200 and 50.0 MHz, respectively, in DMSO-*d*_*6*_ or CDCl_3_ solution using a Bruker Avance II series 200 MHz spectrometer. Column chromatography was performed with Merck silica gel (230 − 400 mesh ASTM) or a Büchi Sepacor chromatography module (prepacked cartridge system). Elemental analyses (C, H, N) were within ± 0.4% of theoretical values and were performed with a VARIO EL III elemental analyzer. The purity of all the tested compounds was > 95%, determined by HPLC (Agilent 1100 series).

Compounds **9a–e** were prepared according to published procedure (Barreca et al. 2022a).

### General procedure for the synthesis of pyrrolo[1,2-h][1,7]naphthyridinones (7a–c) and pyrido[2,3-c]pyrrolo[1,2-a]azepinones (8a, b)

To a solution of enamiketones **9a–e** (2.76 mmol) in anhydrous ethanol (20 mL) phenylsulfonylacetonitrile (Sigma-Aldrich, Schnelldorf, Germany) (0.75 g, 4.14 mmol) was added under nitrogen atmosphere. The reaction mixture was heated under reflux for 24 h. After cooling, the solvent was removed under reduced pressure. The crude product was recrystallized from diethyl ether.

#### 3-(benzenesulfonyl)-5,6-dihydropyrrolo[1,2-h][1,7]naphthyridin-2(1H)-one (7a)

This compound was obtained by reaction of **9a**. Yellow solid; yield: 64%; mp: 317–318 °C; IR cm^−1^: 3119 (NH), 1641 (CO); ^1^H NMR (DMSO-*d*_*6*_, 200 MHz, ppm): *δ* 2.95 (t, 2H, *J* = 6.7 Hz, CH_2_), 4.11 (t, 2H, *J* = 6.7 Hz, CH_2_) 6.22–6.25 (m, 1H, Ar), 7.13–7.16 (m, 2H, Ar), 7.54–7.71 (m, 3H, H-Ar), 7.95–8.00 (m, 2H, Ar), 8.20 (s, 1H, H-4), 12.38 (s, 1H, NH). ^13^C NMR (DMSO-*d*_*6*_, 50 MHz, ppm): *δ* 25.4, 43.4, 106.2, 110.1, 111.7, 121.7, 127.0, 127.8, 128.7, 133.0, 135.5, 140.8, 141.9, 143.7, 157.2. Anal. Calcd. for C_17_H_14_N_2_O_3_S: C, 62.56; H, 4.32; N, 8.58. Found: C, 62.72; H, 4.24; N, 8.41.

#### Ethyl 3-(benzenesulfonyl)-2-oxo-1,2,5,6-tetrahydropyrrolo[1,2-h][1,7]naphthyridine-8-carboxylate (7b)

This compound was obtained by reaction of **9b**. Brown solid; yield: 70%; mp: 145–146 °C; IR cm^−1^: 3365 (NH), 1718 (CO), 1646 (CO); ^1^H NMR (DMSO-*d*_*6*_, 200 MHz, ppm): *δ* 1.29 (t, 3H, *J* = 7.1 Hz, CH_3_), 3.01 (t, 2H, *J* = 7.0 Hz, CH_2_), 4.27 (q, 2H, *J* = 7.1 Hz, CH_2_), 4.54 (t, 2H, *J* = 7.0 Hz, CH_2_), 6.65 (d, 1H, *J* = 4.0 Hz, Ar), 7.16 (d, 1H, *J* = 4.0 Hz, Ar), 7.55–7.73 (m, 3H, Ar), 7.96–8.01 (m, 2H, Ar), 8.29 (s, 1H, H-4), 12.58 (s, 1H, NH); ^13^C NMR (DMSO-*d*_*6*_, 50 MHz, ppm): *δ* 14.2, 24.8, 42.1, 60.3, 108.4, 110.4, 117.6, 118.7, 120.1, 125.5, 128.0, 128.8, 133.3, 138.9, 140.3, 144.1, 157.1, 159.9. Anal. Calcd. for C_20_H_18_N_2_O_5_S: C, 60.29; H, 4.55; N, 7.03. Found: C, 60.17; H, 4.69; N, 6.85.

#### Propan-2-yl 3-(benzenesulfonyl)-2-oxo-1,2,5,6-tetrahydropyrrolo[1,2-h][1,7]naphthyridine-8-carboxylate (7c)

This compound was obtained by reaction of **9c**. White solid; yield: 71%, mp: 332–333 °C; IR: 3125 (NH), 1703 (CO), 1652 (CO) cm^−1^; ^1^H NMR (DMSO-*d*_*6*_, 200 MHz, ppm): *δ* 1.29 (6H, d, *J* = 6.2 Hz, 2 × CH_3_), 3.00 (2H, t, *J* = 6.7 Hz, CH_2_), 4.54 (2H, t, *J* = 6.7 Hz, CH_2_), 5.07–5.13 (1H, m, CH), 6.91 (1H, d, *J* = 4.1 Hz, Ar), 7.14 (1H, d, *J* = 4.1 Hz, Ar), 7.56–7.69 (3H, m, Ar), 7.99 (2H, d, *J* = 7.5 Hz, Ar), 8.29 (1H, s, H-4), 12.57 (1H, s, NH); ^13^C NMR (DMSO-*d*_*6*_, 50 MHz, ppm): *δ* 22.1, 25.3, 42.6, 68.3, 107.5, 110.8, 118.0, 123.9, 126.3, 128.5, 129.2, 133.8 (2 × C), 140.8, 145.8, 149.7, 157.5, 160.0. Anal. Calcd. for C_21_H_20_N_2_O_5_S: C, 61.15; H, 4.89; N, 6.79. Found: C, 61.33; H, 5.02; N, 6.64.

#### Ethyl 3-(benzenesulfonyl)-2-oxo-1,5,6,7-tetrahydro-2H-pyrido[2,3-c]pyrrolo[1,2-a]azepine-9-carboxylate (8a)

This compound was obtained by reaction of **9d**. Yellow solid; yield: 61%, mp: 340–341 °C; IR: 3131 (NH), 1703 (CO), 1652 (CO) cm^−1^; ^1^H NMR (DMSO-*d*_*6*_, 200 MHz, ppm): *δ* 1.29 (3H, t, *J* = 7.1 Hz, CH_3_), 2.23–2.26 (2H, m, CH_2_), 2.32–2.59 (2H, s, CH_2_), 4.27 (2H, q, *J* = 7.1 Hz, CH_3_), 4.38 (2H, t, *J* = 6.1 Hz, CH_2_), 6.64 (1H, d, *J* = 4.2 Hz, Ar), 6.94 (1H, d, *J* = 4.2 Hz, Ar), 7.57–7.70 (3H, m, Ar), 8.00 (2H, d, *J* = 6.9 Hz, Ar), 8.33 (1H, s, H-4), 12.54 (1H, s, NH); ^13^C NMR (DMSO-*d*_*6*_, 50 MHz, ppm): *δ* 14.7, 27.2, 31.8, 44.0, 60.6, 112.7, 117.0, 117.7, 125.7, 126.5, 128.7, 129.3, 133.3, 133.8, 140.7, 145.2, 146.0, 157.6, 160.5. Anal. Calcd. for C_21_H_20_N_2_O_5_S: C, 61.15; H, 4.89; N, 6.79. Found: C, 61.02; H, 4.69; N, 6.91.

#### Propan-2-yl 3-(benzenesulfonyl)-2-oxo-1,5,6,7-tetrahydro-2H-pyrido[2,3-c]pyrrolo[1,2-a]azepine-9-carboxylate (8b)

This compound was obtained by reaction of **9e**. White solid; yield: 54%, mp: 237–238 °C; IR: 3131 (NH), 1697 (CO), 1646 (CO) cm^−1^; ^1^H NMR (DMSO-*d*_*6*_, 200 MHz, ppm): *δ* 1.30 (6H, d, *J* = 6.1 Hz, 2 × CH_3_), 2.12–2.34 (2H, m, CH_2_), 2.42–2.51 (2H, m, CH_2_), 4.29–4.43 (2H, m, CH_2_), 5.04–5.16 (1H, m, CH), 6.63 (1H, d, *J* = 4.1 Hz, Ar), 6.91 (1H, d, *J* = 4.1 Hz, Ar), 7.57–7.73 (3H, m, Ar), 8.01 (2H, d, *J* = 6.9 Hz, Ar), 8.33 (1H, s, H-4), 12.53 (1H, s, NH); ^13^C NMR (DMSO-*d*_*6*_, 50 MHz, ppm): *δ* 21.7, 26.7, 31.3, 43.6, 67.6, 102.8, 112.1, 116.4, 121.4, 125.6, 128.2, 128.8, 133.4, 137.0, 140.1, 145.5, 155.7, 157.2, 159.7. Anal. Calcd. for C_22_H_22_N_2_O_5_S: C, 61.96; H, 5.20; N, 6.57. Found: C, 62.22; H, 5.05; N, 6.68.

### General procedure for the synthesis of N-methyl derivatives (7d–f and 8c, d) and O-methyl derivatives (7g–i and 8e, f)

To a solution of derivatives **7a–c** and **8a, b** (15 mmol) in anhydrous DMF (20 mL), NaH (Sigma-Aldrich, Schnelldorf, Germany) (0.64 g, 16 mmoli) was added at 0 °C and the reaction mixture was stirred at room temperature for 6 h. Then, iodomethane (Sigma-Aldrich, Schnelldorf, Germany) (16 mmol) was added at 0 °C and the reaction mixture was stirred at room temperature for 24 h. The reaction mixture was poured onto crushed ice. The precipitate was filtered off and dried, in absence the solution was extracted with ethyl acetate (3 × 30 mL). The organic layer was dried over Na_2_SO_4_ and the solvent was removed under reduced pressure. The crude product, containing *N*-methyl substituted derivatives **7d–f** and **8c, d** and *O*-methyl **7g–i** and **8e, f**, was purified by column chromatography (DCM/AcOEt 95:5).

#### 1-Methyl-3-(benzenesulfonyl)-5,6-dihydropyrrolo[1,2-h][1,7]naphthyridin-2(1H)-one (7d)

This compound was obtained by reaction of **7a**. Yellow solid; yield: 57%, mp: 268–269 °C; IR: 1658 (CO) cm^−1^; ^1^H NMR (CDCl_3_, 200 MHz, ppm): *δ* 3.11 (2H, t, *J* = 6.7 Hz, CH_2_), 3.95 (3H, s, CH_3_), 4.14 (2H, t, *J* = 6.7 Hz, CH_2_), 6.31–6.35 (1H, m, Ar), 6.81–6.91 (1H, m, Ar), 6.92–6.95 (1H, m, Ar), 7.46–7.58 (3H, m, Ar), 8.11–8.16 (2H, m, Ar), 8.21 (1H, s, H-4); ^13^C NMR (CDCl_3_, 50 MHz, ppm): *δ* 29.7, 34.6, 44.6, 108.2, 110.0, 115.7, 122.0, 122.5, 126.1, 128.6, 128.8, 133.0, 140.3, 141.5, 144.5, 157.8. Anal. Calcd. for C_18_H_16_N_2_O_3_S: C, 63.51; H, 4.74; N, 8.23. Found: C, 63.62; H, 4.55; N, 8.07.

#### Ethyl 3-(benzenesulfonyl)-1-methyl-2-oxo-1,2,5,6-tetrahydropyrrolo[1,2-h][1,7]naphthyridine-8-carboxylate (7e)

This compound was obtained by reaction of **7b**. Yellow solid; yield: 53%, mp: 250–251 °C; IR: 1709 (CO), 1658 (CO) cm^−1^; ^1^H NMR (CDCl_3_, 200 MHz, ppm): *δ* 1.38 (3H, t, *J* = 7.1 Hz, CH_3_), 2.93 (2H, t, *J* = 6.4 Hz, CH_2_), 3.77 (3H, s, CH_3_), 4.35 (2H, q, *J* = 7.1 Hz, CH_2_), 4.68 (2H, t, *J* = 6.4 Hz, CH_2_), 6.76 (1H, d, *J* = 4.4 Hz, Ar), 7.03 (1H, d, *J* = 4.4 Hz, Ar), 7.47–7.64 (3H, m, 3H-Ar), 8.12–8.18 (2H, m, 2H-Ar), 8.27 (1H, s, H-4); ^13^C NMR (CDCl_3_, 50 MHz, ppm): *δ* 14.4, 27.7, 35.2, 41.8, 60.9, 110.7, 113.9, 117.3, 125.4, 125.5, 127.1, 128.6, 129.0, 133.3, 139.9, 141.5, 143.1, 157. 8, 160.5. Anal. Calcd. for C_21_H_20_N_2_O_5_S: C, 61.15; H, 4.89; N, 6.79. Found: C, 60.95; H, 5.02; N, 6.66.

#### Propan-2-yl 3-(benzenesulfonyl)-1-methyl-2-oxo-1,2,5,6-tetrahydropyrrolo[1,2-h][1,7]naphthyridine-8-carboxylate (7f)

This compound was obtained by reaction of **7c**. Yellow solid; yield: 55%, mp: 207–208 °C; IR: 1703 (CO), 1658 (CO) cm^−1^; ^1^H NMR (CDCl_3_, 200 MHz, ppm): *δ* 1.36 (6H, d, *J* = 6.2 Hz, 2 × CH_3_), 2.92 (2H, t, *J* = 7.2 Hz, CH_2_), 3.77 (3H, s, CH_3_), 4.68 (2H, t, *J* = 7.2 Hz, CH_2_), 5.18–5.24 (1H, m, CH), 6.75 (1H, d, *J* = 4.4 Hz, Ar), 7.01 (1H, d, *J* = 4.4 Hz, Ar), 7.47–7.63 (3H, m, Ar), 8.11–8.17 (2H, m, Ar), 8.27 (1H,s, H-4); ^13^C NMR (CDCl_3_, 50 MHz, ppm): *δ* 21.9, 27.7, 35.1, 41.8, 68.5, 110.7, 113.8, 117.2, 125.4, 125.), 126.9, 128.6, 129.1, 133.3, 139.9, 141.5, 143.2, 157.8, 160.1. Anal. Calcd. for C_22_H_22_N_2_O_5_S: C, 61.96; H, 5.20; N, 6.57. Found: C, 62.09; H, 5.34; N, 6.29.

#### 2-methoxy-3-(phenylsulfonyl)-5,6-dihydropyrrolo[1,2-h][1,7]naphthyridine (7g)

This compound was obtained by reaction of **7a**. White solid; yield: 37%; mp: 210–211 °C; ^1^H NMR (CDCl_3_, 200 MHz, ppm): *δ* 3.11 (2H, t, *J* = 6.6 Hz, CH_2_), 3.95 (3H, s, CH_3_), 4.12 (2H, t, *J* = 6.6 Hz, CH_2_), 6.25–6.28 (1H, m, Ar), 6.81 (1H, s, Ar), 6.88–6.90 (1H, m, Ar), 7.44- 7.61 (3H, m, Ar), 7.97–8.01 (2H, m, Ar), 8.15 (1H, s, H-4); ^13^C NMR (CDCl_3_, 50 MHz, ppm): *δ* 27.4, 43.9, 53.9, 108.2, 110.0, 110.3, 117.5, 118.8, 124.4, 128.4, 128.6, 129.3, 133.0, 138.3, 141.1, 150.4, 159.2. Anal. Calcd. for C_18_H_16_N_2_O_3_S: C, 63.51; H, 4.74; N, 8.23. Found: C, 63.43; H, 4.88; N, 7.99.

#### Ethyl 3-(benzenesulfonyl)-2-methoxy-5,6-dihydropyrrolo[1,2-h][1,7]naphthyridine-8-carboxylate (7h)

This compound was obtained by reaction of **7b**. Light yellow solid; yield: 35%, mp: 157–158 °C; IR: 1697 (CO) cm^−1^; ^1^H NMR (CDCl_3_, 200 MHz, ppm): *δ* 1.37 (3H, t, *J* = 7.1 Hz, CH_3_), 3.13 (2H, t, *J* = 6.9 Hz, CH_2_), 3.96 (3H, s, CH_3_), 4.32 (2H, q, *J* = 7.1 Hz, CH_2_), 4.67 (2H, t, *J* = 6.9 Hz, CH_2_), 6.88 (1H, d, *J* = 4.1 Hz, Ar), 7.01 (1H, d, *J* = 4.1 Hz, Ar), 7.46–7.64 (3H, m, 3H-Ar), 7.98–8.03 (2H, m, Ar), 8.23 (1H, s, H-4); ^13^C NMR (CDCl_3_, 50 MHz, ppm): *δ* 14.4, 26.9, 42.2, 54.0, 60.4, 109.5, 118.4, 119.2, 121.0, 124.9, 128.5, 128.7, 133.3, 134.6, 138.5, 140.6, 149.2, 159.11, 161.1. Anal. Calcd. for C_21_H_20_N_2_O_5_S: C, 61.15; H, 4.89; N, 6.79. Found: C, 61.31; H, 4.76; N, 6.90.

#### Propan-2-yl 3-(benzenesulfonyl)-2-methoxy-5,6-dihydropyrrolo[1,2-h][1,7]naphthyridine-8-carboxylate (7i)

This compound was obtained by reaction of **7c**. Yellow solid; yield: 44%, mp: 168–169 °C; IR: 1692 (CO) cm^−1^; ^1^H NMR (CDCl_3_, 200 MHz, ppm): *δ* 1.35 (6H, d, *J* = 6.2 Hz, 2 × CH_3_), 3.31 (2H, t, *J* = 6.9 Hz, CH_2_), 3.97 (3H, s, CH_3_), 4.67 (2H, t, *J* = 6.9 Hz, CH_2_), 5.13–5.26 (1H, m, CH), 6.88 (1H, d, *J* = 4.1 Hz Ar), 6.99 (1H, d, *J* = 4.1 Hz, Ar), 7.47–7.63 (3H, m, Ar), 8.03 (2H, d, *J* = 6.6 Hz Ar), 8.22 (1H,s, H-4); ^13^C NMR (CDCl_3_, 50 MHz, ppm): *δ* 22.0, 26.9, 42.2, 54.0, 67.9, 109.4, 118.2, 119.1., 120.9, 125.4, 128.5, 128.7, 133.3, 134.5, 138.5, 140.7, 149.2, 159.1, 160.7. Anal. Calcd. for C_22_H_22_N_2_O_5_S: C, 61.96; H, 5.20; N, 6.57. Found: C, 61.82; H, 5.41; N, 6.28.

#### Ethyl 3-(benzenesulfonyl)-1-methyl-2-oxo-1,5,6,7-tetrahydro-2H-pyrido[2,3-c]pyrrolo[1,2-a]azepine-9-carboxylate (8c)

This compound was obtained by reaction of **8a**. Yellow solid; yield: 55%, mp: 238–239 °C; IR: 1697 (CO), 1658 (CO) cm^−1^; ^1^H NMR (CDCl_3_, 200 MHz, ppm): *δ* 1.39 (3H, t, *J* = 7.1 Hz, CH_3_), 2.05–2.19 (2H, m, CH_2_), 2.37–2.62 (2H, m, CH_2_), 3.60 (3H, s, CH_3_), 4.34(2H, q, *J* = 7.1 Hz, CH_2_), 5.35–5.45 (2H, m, CH_2_), 6.37 (1H, d, *J* = 4.2 Hz, Ar), 7.04 (1H, d, *J* = 4.2 Hz, Ar), 7.49–7.65 (3H, m, Ar), 8.11–8.18 (2H, m, Ar), 8.28 (1H, s, H-4); ^13^C NMR (CDCl_3_, 50 MHz, ppm): *δ* 14.4, 28.3, 31.9, 35.3, 43.3, 60.6, 112.6, 116.3, 116.8, 124.4, 127.6, 128.6, 129.1, 130.6, 133.4, 139.7, 143.3, 145.8, 157.6, 160.7. Anal. Calcd. for C_22_H_22_N_2_O_5_S: C, 61.96; H, 5.20; N, 6.57. Found: C, 62.11; H, 5.07; N, 6.69.

#### Propan-2-yl 3-(benzenesulfonyl)-1-methyl-2-oxo-1,5,6,7-tetrahydro-2H-pyrido[2,3-c]pyrrolo[1,2-a]azepine-9-carboxylate (8d)

This compound was obtained by reaction of **8b**. Yellow solid; yield: 51%, mp: 253–254 °C; IR: 1697 (CO), 1658 (CO) cm^−1^; ^1^H NMR (CDCl_3_, 200 MHz, ppm): *δ* 1.37 (6H, d, *J* = 6.2 Hz, 2 × CH_3_), 2.08–2.20 (2H, m, CH_2_), 2.41–2.61 (2H, m, CH_2_), 3.60 (3H, s, CH_3_), 5.15–5.44 (3H, m, CH_2_ and CH), 6.35 (1H, d, *J* = 4.1 Hz, Ar), 7.02 (1H, d, *J* = 4.1 Hz, Ar), 7.54–7.62 (3H, m, Ar), 8.14–8.19 (2H, m, Ar), 8.29 (1H,s, H-4); ^13^C NMR (CDCl_3_, 50 MHz, ppm): *δ* 22.0, 28.4, 31.8, 35.3, 43.3, 68.2, 112.5, 116.3, 117.8, 124.9, 127.7, 128.6, 129.2, 130.5, 133.4, 139.7, 143.3, 145.9, 157.6, 160.2. Anal. Calcd. for C_23_H_24_N_2_O_5_S: C, 62.71; H, 5.49; N, 6.36. Found: C, 62.57; H, 5.22; N, 6.58.

#### Ethyl 3-(benzenesulfonyl)-2-methoxy-6,7-dihydro-5H-pyrido[2,3-c]pyrrolo[1,2-a]azepine-9-carboxylate (8e)

This compound was obtained by reaction of **8a**. White solid; yield: 43%, mp: 157–158 °C; IR: 1697 cm^−1^; ^1^H NMR (CDCl_3_, 200 MHz, ppm): *δ* 1.38 (3H, t, *J* = 7.1 Hz, CH_3_), 2.31–2.45 (2H, m, CH_2_), 2.70 (2H, t, *J* = 7.0 Hz, CH_2_), 3.96 (3H, s, CH_3_), 4.27–4.45 (4H, m, 2 × CH_2_), 6.62 (1H, d, *J* = 4.1 Hz, Ar), 7.01 (1H, d, *J* = 4.1 Hz, Ar), 7.49–7.65 (3H, m, Ar), 8.01–8.06 (2H, m, Ar), 8.27 (1H,s, H-4); ^13^C NMR (CDCl_3_, 50 MHz, ppm): *δ* *δ* 14.4, 28.7, 31.5, 43.8, 51.1, 60.3, 111.0, 117.4, 121.6, 125.0, 126.5, 128.6, 128.7, 133.4, 140.0, 140.5, 140.6, 153.4, 158.4, 161.1. Anal. Calcd. for C_22_H_22_N_2_O_5_S: C, 61.96; H, 5.20; N, 6.57. Found: C, 61.83; H, 5.01; N, 6.75.

#### Propan-2-yl 3-(benzenesulfonyl)-2-methoxy-6,7-dihydro-5H-pyrido[2,3-c]pyrrolo[1,2-a]azepine-9-carboxylate (8f)

This compound was obtained by reaction of **8b**. White solid; yield: 46%, mp: 211–212 °C; IR: 1696 (CO) cm^−1^; ^1^H NMR (CDCl_3_, 200 MHz, ppm): *δ* 1.35 (6H, d, *J* = 6.2 Hz, 2 × CH_3_), 2.35–2.44 (2H, m, CH_2_), 2.64–2.73 (2H, m, CH_2_), 3.95 (3H, s, CH3), 4.41 (2H, t, *J* = 6.5 Hz, CH_2_), 5.13–5.26 (1H, m, CH), 6.62 (1H, d, *J* = 4.1 Hz, Ar), 7.00 (1H, d, *J* = 4.1 Hz, Ar), 7.49–7.65 (3H, m, Ar), 8.0 (2H, d, *J* = 6.8 Hz, Ar), 8.27 (1H,s, H-4); ^13^C NMR (CDCl_3_, 50 MHz, ppm): *δ* 22.1, 28.7, 31.5, 43.7, 54.1, 67.7, 110.9, 117.3, 121.6, 125.5, 126.5, 128.6, 128.7, 133.4, 139.9, 140.5, 153.4, 158.4, 159.8, 160.7. Anal. Calcd. for C_23_H_24_N_2_O_5_S: C, 62.71; H, 5.49; N, 6.36. Found: C, 62.88; H, 5.61; N, 6.24.

### General procedure for the synthesis of 9-bromo-pyrrolo[1,2-h][1,7]naphthyridine (7j,k) and 10-bromo-pyrido[2,3-c]pyrrolo[1,2-a]azepine (8g, h)

To a solution of derivative **7b, c** and **8a, b** (0.22 mmol) in anhydrous DCM (20 ml), Br_2_ (Sigma-Aldrich, Schnelldorf, Germany) (0.44 mmol, 0.02 mL) was added at 0 °C and the reaction mixture was stirred at room temperature for 24 h. Then the reaction mixture was evaporated under reduced pressure. The crude product was recrystallized from diethyl ether.

#### Ethyl 3-(benzenesulfonyl)-8-bromo-2-oxo-1,2,5,6-tetrahydropyrrolo[1,2-h][1,7]naphthyridine-8-carboxylate (7j)

This compound was obtained by reaction of **7b**. Yellow solid; yield: 63%; mp: 196–197 °C; IR: 3342 (NH), 1700 (CO), 1669 (CO) cm^−1^; ^1^H NMR (DMSO-*d*_*6*_, 200 MHz, ppm): *δ* 1.37 (3H, t, *J* = 7.1 Hz, CH_3_), 2.99 (2H, t, *J* = 7.0 Hz, CH_2_), 4.33 (2H, q, *J* = 7.1 Hz, CH_2_), 4.67 (2H, t, *J* = 7.0 Hz, CH_2_), 7.03 (1H, s, Ar), 7.49–7.65 (3H, m, Ar), 8.09–8.17 (2H, m, Ar), 8.31 (1H, s, H-4), 10.02 (1H, s, NH); ^13^C NMR (DMSO-*d*_*6*_, 50 MHz, ppm): *δ* 14.3, 23.8, 42.8, 61.3, 97.5, 108.2, 120.8, 122.3, 126.2, 127.9, 128.7, 129.0, 133.6, 138.8, 139.6, 143.8, 156.0, 159.6. Anal. Calcd. for C_20_H_17_BrN_2_O_5_S: C, 50.32; H, 3.59; N, 5.87. Found: C, 50.16; H, 3.72; N, 5.65.

#### Propan-2-yl 3-(benzenesulfonyl)-8-bromo-2-oxo-1,2,5,6-tetrahydropyrrolo[1,2-h][1,7]naphthyridine-8-carboxylate (7k)

This compound was obtained by reaction of **7c**. White solid; yield: 69%, mp: 174–175 °C; IR: 3336 (NH), 1705 (CO), 1669 (CO) cm^−1^; ^1^H NMR (CDCl_3_, 200 MHz, ppm): *δ* 1.40 (6H, d, *J* = 6.1 Hz, 2 × CH_3_), 3.06 (2H, s, CH_2_), 4.71 (2H, s, CH_2_), 5.20–5.28 (1H, m, CH), 7.02 (1H, s, Ar), 7.55–7.63 (3H, m, Ar), 8.17–8.19 (2H, m, Ar), 8.37 (1H, s, H-4), 9.25 (1H, s, NH); ^13^C NMR (CDCl_3_, 50 MHz, ppm): *δ* 22.1, 26.3, 43.7, 69.2, 98.0, 102.7, 109.3, 120.9, 122.4, 124.9, 126.8, 129.1, 129.5, 133.7, 144.6, 156.3, 158.7, 159.1. Anal. Calcd. for C_21_H_19_BrN_2_O_5_S: C, 51.33; H, 3.90; N, 5.70. Found: C, 51.09; H, 4.14; N, 5.41.

#### Ethyl 3-(benzenesulfonyl)-9-bromo-2-oxo-1,5,6,7-tetrahydro-2H-pyrido[2,3-c]pyrrolo[1,2-a]azepine-9-carboxylate (8g)

This compound was obtained by reaction of **8a**. White solid; yield: 90%, mp: 172–173 °C; IR: 3399 (NH), 1705 (CO), 1652 (CO) cm^−1^; ^1^H NMR (CDCl_3_, 200 MHz, ppm): *δ* 1.33 (3H, t, *J* = 7.1 Hz, CH_3_), 2.18–2.21 (2H, m, CH_2_), 2.38–2.46 (2H, m, CH_2_), 4.21–4.38 (4H, m, 2 × CH_2_), 7.05 (1H, s, Ar), 7.58–7.75 (3H, m, Ar), 8.02 (2H, d, *J* = 6.9 Hz, Ar), 8.37 (1H, s, H-4), 12.48 (1H, s, NH); ^13^C NMR (CDCl_3_, 50 MHz, ppm): *δ* 14.0, 26.0, 31.4, 46.7, 61.1, 98.0, 106.7, 119.2, 123.0, 124.6, 128.3, 128.8, 134.0, 140.3, 140.4, 147.9, 157.3, 159.1, 159.7. Anal. Calcd. for C_21_H_19_BrN_2_O_5_S: C, 51.33; H, 3.90; N, 5.70. Found: C, 51.51; H, 3.73; N, 5.89.

#### Propan-2-yl 3-(benzenesulfonyl)-9-bromo-2-oxo-1,5,6,7-tetrahydro-2H-pyrido[2,3-c]pyrrolo[1,2-a]azepine-9-carboxylate (8h)

This compound was obtained by reaction of **8b**. White solid; yield: 84%, mp: 167–168 °C; IR: 3422 (NH), 1702 (CO), 1646 (CO) cm^−1^; ^1^H NMR (DMSO-*d*_*6*_, 200 MHz, ppm): *δ* 1.28–1.35 (6H, m, 2 × CH_3_), 2.18–2.41 (2H, m, CH_2_), 2.44 (2H, s, CH_2_), 4.16 (2H, s, CH_2_), 5.03–5.15 (1H, m, CH), 7.00 (1H, s, H-Ar), 7.58–7.72 (3H, m, 3 × H-Ar), 8.01 (2H, d, 2H-Ar), 8.37 (1H, s, 1H-Ar), 12.11–12.81 (1H, m, NH); ^13^C NMR (DMSO-*d*_*6*_, 50 MHz, ppm): *δ* 22.0, 27.6, 31.8, 44.8, 68.8, 98.5, 107.5, 119.4, 119.6, 126.7, 127.7, 128.6, 129.0, 129.1, 134.0, 139.1, 142.2, 146.8, 159.2. Anal. Calcd. for C_22_H_21_BrN_2_O_5_S: C, 52.28; H, 4.19; N, 5.54. Found: C, 52.42; H, 4.01; N, 5.67.

## Biology

All tested compounds were dissolved in the minimum DMSO amount prior to cell culture testing. A calculated amount of the stock DMSO solution was added to the cell culture media to reach a final maximum DMSO concentration of 0.5%, which had no discernable effects on cell viability.

### Light exposure

The irradiation device used for the irradiation of cell cultures during the assays was a black box equipped with two Philips UVA lamps with an irradiance of 17 mW cm^−2^ and a centered emission peak at 365 nm; UVA irradiance was measured using a Waldmann Variocontrol radiometer, equipped with a UVA sensor. The box was maintained at room temperature inside with a fan and the multiwell plates were placed onto a water-refrigerated metal block.

### Cell cultures

Human triple negative (TNBC) breast MDA-MB-231 carcinoma cells were obtained from American Type Culture Collection (ATCC, Rockville, MD, USA). Human embryonic kidney HEK293 cells were obtained from the European Collection of Cell Cultures (ECACC, Salisbury, UK). Cell lines were maintained in the logarithmic phase at 37 °C in a 5% CO_2_ atmosphere using the following culture media containing 10% foetal calf serum (Euroclone, Milan, Italy), antibiotics (50 units/mL penicillin and 50 μg/mL streptomycin) and 2 mM l-glutamine: (i) RPMI-1640 medium (Euroclone, Milan, Italy) for MDA-MB-231 4 cells; (ii) DMEM (Euroclone, Milan, Italy) for HEK293 cells.

### MTT assay

Cells (5–8 × 10^3^ cells/well, dependent upon the growth characteristics of the cell line) were seeded in 96-well microplates in growth medium (100 μL). After 24 h, the medium was removed and replaced with a fresh one containing the compound to be studied at the appropriate concentration. Triplicate cultures were established for each treatment. For photocytotoxicity studies, after 1 h of incubation the medium was replaced with PBS and cells were treated with 2.0 J cm^−2^ of UVA light. After irradiation, PBS was replaced with complete proliferation media. For pre-irradiation studies, instead, compounds in PBS were irradiated with 2.0 J cm^−2^ of UVA light and subsequently cells were treated with pre-irradiated solutions. Following 24 h, each well was treated with 10 μL of a 5 mg/mL MTT saline solution, and following 5 h of incubation, 100 μL of a sodium dodecyl sulfate (SDS) solution in HCl 0.01 M were added. After an overnight incubation, cell growth inhibition was detected by measuring the absorbance of each well at 570 nm using a Bio-Rad 680 microplate reader. Mean absorbance for each drug dose was expressed as a percentage of the control untreated well absorbance and plotted against drug concentration. IC_50_ values, the drug concentrations that reduce the mean absorbance at 570 nm to 50% of those in the untreated control wells, were calculated by the four-parameter logistic (4-PL) model. Evaluation was based on means from at least four independent experiments.

### Cell death induction

MDA-MB-231 cells were seeded into 8-well tissue-culture slides (BD Falcon, Bedford, MA, USA) at 5 × 10^4^ cells/well (0.8 cm^2^). After 24 h, the cells were washed twice with PBS and, following 24 h of treatment with IC_50_ doses of the tested compound, cells were stained for 5 min with 10 µg/mL of Hoechst 33258 (20-(4-hydroxyphenyl)-5-(4-methyl-1-piperazinyl)-2,50-bi-1H-benzimidazole trihydrochloride hydrate, Sigma-Aldrich, St.Louis, MI, USA) or propidium iodide (PI, Sigma-Aldrich, St.Louis, MI, USA) in PBS. Samples were examined at  × 5 and × 40 magnification in a Zeiss LSM 800 confocal microscope using the Zeiss ZEN 2.3 software system.

### Mitochondrial Membrane Potential (ΔΨ)

The ΔΨ was assayed using the Mito-ID® Membrane Potential Kit according to the manufacturer’s instructions (Enzo Life Sciences, Farmingdale, NY, USA) as previously described. Briefly, MDA-MB-231 cells (8 × 10^3^ per well) were seeded in 96-well plates; after 24 h, cells were washed with PBS and loaded with Mito-ID Detection Reagent for 30 min at 37 °C in the dark. Afterwards, cells were incubated with increasing concentrations of tested compounds for 90 min and after that exposed to UVA light (2.0 J/cm^2^). CCCP (Carbonyl cyanide 3-chlorophenyl-hydrazone) was used as positive control. Fluorescence intensity was estimated using a VICTOR X3 (PerkinElmer, USA) plate reader at 490 (excitation) and 590 nm (emission).

### ROS production

The production of ROS was measured in MDA-MB-231 cells (10^4^ per well) grown for 24 h in a 96-well plate in RPMI medium without phenol red (Merk, Germany). Cells were then washed with PBS and loaded with 10 μM 5-(and-6)-chloromethyl-2′,7′-dichlorodihydrofluorescein diacetate acetyl ester (CM–H_2_DCFDA) (Molecular Probes-Invitrogen, Eugene, OR) for 45 min, in the dark. Afterwards, cells were washed with PBS and incubated with tested compounds (25 µM) for 90 min and after that exposed to UVA light (2.0 J/cm^2^). Fluorescence increase was estimated utilizing the wavelengths of 485 nm (excitation) and 527 nm (emission) in a VICTOR X3 (PerkinElmer, USA) plate reader. Antimycin (3 μM, Merk), a potent inhibitor of Complex III in the electron transport chain, was used as positive control.

### Quantification of thiols

The MDA-MB-231 cells (2 × 10^5^) were seeded in a six-well plate in growth medium (4 mL). After 24 h, cells were incubated with tested compounds (25 µM) for 90 min, and after that exposed to UVA light (2.0 J/cm^2^). Subsequently, after 24 h, the thiol content was measured as previously described (Rigobello et al. 2009).

### Generation of active oxygen species

*Singlet oxygen determination*. Samples containing the compounds under examination (2.2 × 10^–5^ M), *p*-nitrosodimethylaniline (4 × 10^–5^ M) and imidazole (4 × 10^–5^ M) in 0.02 M phosphate buffer (pH 7.3) were irradiated with increasing UVA doses and their absorbance at 440 nm was then measured (Kraljic and El Mohsni 1978). The data were expressed as percentage of RNO bleaching.

#### Superoxide radical determination

Samples containing the compounds under examination (10^–5^ M) and nitroblue tetrazolium (1.6 × 10^–4^ M) in 10 mM carbonate buffer (pH 10) were irradiated with increasing UVA doses, and their absorbance at 560 nm was measured (Pathak and Joshi 1984).

### UVA Photostability of the compounds

Samples containing each compound (2.2 × 10^− 5^ M) dissolved in PBS (0.04 M) were irradiated with increasing UVA doses from 0 to 20 J/cm^2^ (0.2, 5, 10, 15 and 20 J/cm^2^), and their absorption spectrum was measured, with a Cary 50 Scan UV–Visible Spectrophotometer.

### Cellular biodistribution studies

MDA-MB-231 cells were seeded into 4-well tissue-culture slides (BD Falcon, Bedford, MA, USA) at 2.5 × 10^4^ cells/well (0.4 cm^2^). After 24 h, after 1 h of incubation the medium was replaced with PBS and cells were treated with 2.0 J cm^−2^ of UVA light. After irradiation, PBS was replaced with complete proliferation media. Samples were examined at 20 × magnification in a Zeiss LSM 800 confocal microscope using the Zeiss ZEN 2.3 software system.

### Statistical analysis

Data are expressed as mean ± the standard deviation (SD) of the mean. The difference between groups was evaluated using analysis of variance (ANOVA) with Graphpad Prism 9.0.

## Results

### Chemistry

To access the pyrrolo[1,2-*h*][1,7]naphthyridinone (**7**) and pyrido[2,3-*c*]pyrrolo[1,2-*a*]azepinone (**8**) systems we started our synthesis from ketones 6,7-dihydroindolizin-8(5*H*)-one (n = 1) and 5,6,7,8-tetrahydro-9*H*-pyrrole[1,2-*a*]azepine-9-one (n = 2) respectively, which were properly prepared according to our methods (Barreca et al. 2022a).

**Scheme 1:**
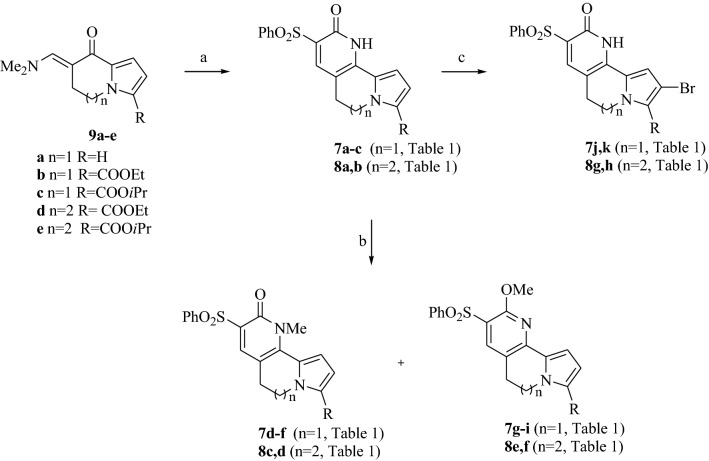
Synthesis of pyrrolo[1,2-*h*][1,7]naphthyridinones (**7**) and pyrido[2,3-*c*]pyrrolo[1,2-*a*]azepinones (**8**). Reagents and conditions: (*a*) PhSO_2_CH_2_CN, ethanol, reflux, 24 h, 54–71%; (*b*) NaH, DMF, 0 °C to rt, 6 h, then iodomethane, 0 °C to rt, 24 h, 35–57%; (*c*) Br_2_, DCM, 0 °C to rt, 24 h, 63–90%

The latter compounds were reacted with an excess of *N,N*-dimethylformamide dimethylacetal (DMFDMA) in *N,N*-dimethylformamide (DMF) to yield enaminoketones **9a–e** in good yields (56–87%) (Scheme 1). These kinds of intermediates have been widely used by us, as they are highly reactive and prone to react with dinucleophyles furnishing several opportunities of annelation. In particular, by reaction with cyanomethylene compounds, the pyridine-2-one ring closure can be achieved. We chose to react enaminoketones **9** with phenylsulfonylacetonitrile, as the phenylsulfonyl group demonstrated crucial for the activity in our previous studies, leading to the desired tricyclic derivatives **7a–c** and **8a, b** (54–71%) (Scheme 1, Table 1) which were further subjected to methylation at the pyridine ring using iodomethane and NaH as the base in DMF, allowing the isolation of the corresponding *N-*methyl **7d–f** and **8c, d** (51–57%) and *O*-methyl substituted **7g–i** and **8e, f** (35–46%) derivatives from the same reaction mixtures (Scheme 1, Table 1). Additionally, compounds **7a–c** and **8a, b** were subjected to bromination of the pyrrole ring with the aim of evaluating the effect of the presence of bromine atom, usually responsible of the so called “heavy atom effect”, on cyto- and phototoxicity. Reactions were carried out using bromine as brominating agent, leading to pyrrolonaphthyridinones **7j, k** and pyridopyrroloazepinones **8g, h** (63–90%) (Scheme 1, Table 1). From the reaction of derivative **7a**, it was not possible to isolate the desired bromo derivative as pure product for biological screenings.

**Table 1:** Pyrrolo[1,2-*h*][1,7]naphthyridinones **7a–k** and pyrido[2,3-*c*]pyrrolo[1,2-*a*]azepinones **8a–h**

CDP	n	R	Yield	CDP	n	R	Yield
**7a**	1	H	64%	**7k**	1	COO*i*Pr	69%
**7b**	1	COOEt	70%	**8a**	2	COOEt	61%
**7c**	1	COO*i*Pr	71%	**8b**	2	COO*i*Pr	54%
**7d**	1	H	57%	**8c**	2	COOEt	55%
**7e**	1	COOEt	53%	**8d**	2	COO*i*Pr	51%
**7f**	1	COO*i*Pr	55%	**8e**	2	COOEt	43%
**7g**	1	H	37%	**8f**	2	COO*i*Pr	46%
**7h**	1	COOEt	35%	**8g**	2	COOEt	90%
**7i**	1	COO*i*Pr	44%	**8h**	2	COO*i*Pr	84%
**7j**	1	COOEt	63%				

## Biology

### Cytotoxicity and photocitotoxicity profiles

The new synthetized pyrrolo[1,2-*h*][1,7]naphthyridinones **7** and pyrido[2,3-*c*]pyrrolo[1,2-*a*]azepinones **8** were screened for their cytotoxicity (in the dark) and photocitotoxicity (upon UVA light irradiation at 2.0 J/cm^2^) profile on human TNBC cells, MDA-MB-231. The cytotoxicity/photocytotoxicity parameters, expressed in terms of IC_50_ values obtained by MTT test after 24 h of drug-exposure, are listed in Table 2.

**Table 2:** Cytotoxic and photocytotoxic activities of pyrrolo[1,2-*h*][1,7]naphthyridinones (**7a–k**) and pyrido[2,3-*c*]pyrrolo[1,2-*a*]azepinones (**8a–h**) against human TNBC MDA-MB-231 cell line

Compound	Dark IC_50_ (µM) ± S.D	UVA (2.0 J/cm^2^) IC_50_ (µM) ± S.D
**7a**	> 50	> 50
**7b**	> 50	21.0 ± 0.4
**7c**	> 50	> 50
**7d**	> 50	> 50
**7e**	> 50	> 50
**7f**	> 50	> 50
**7g**	> 50	> 50
**7h**	> 50	4.4 ± 0.5
**7i**	> 50	7.9 ± 0.5
**7j**	> 50	23.8 ± 1.1
**7k**	> 50	19.8 ± 1.4
**8a**	> 50	> 50
**8b**	> 50	> 50
**8c**	> 50	24.9 ± 2.8
**8d**	> 50	> 50
**8e**	> 50	15.3 ± 1.7
**8f**	> 50	23.7 ± 1.5
**8g**	> 50	22.0 ± 1.2
**8h**	> 50	> 50

Cells (8 × 10^3^ mL^−1^) were pre-treated for 1 h increasing concentrations of the tested compounds, irradiated with UVA light (2.0 J/cm^2^) in the case of UVA irradiated samples, and recovered for total 24 h. The cytotoxicity was assessed by the MTT test. IC_50_ values were calculated by a four-parameter logistic model 4-PL (P < 0.05)

*SD* standard deviation, *ND* not detected

Interestingly, all compounds were completely inactive in the dark and some of them behaved as pure PSs, being effective only upon irradiation. Within the pyrrolo[1,2-*h*][1,7]naphthyridinone series, derivatives **7b** and **7h–k** showed a detectable cytotoxicity and, in particular, compound **7h** and **7i** elicited IC_50_ values in the low micromolar range (IC_50_ 4.4 and 7.9 μM, respectively). Considering the pyrido[2,3-*c*]pyrrolo[1,2-*a*]azepinone series, however, compounds **8c** and **8e–g** were less effective in inhibiting cancer cell viability upon irradiation, with IC_50_ values in the micromolar range (IC_50_ 15.3–24.9 μM). It is of worth noting that the *O*-methyl COOEt substituted derivatives **7h** and **8e** were the most effective compounds among the two series.

In addition, as one of the main drawbacks of chemotherapeutics is the undesired cytotoxic effect toward non-cancerous cells, we also evaluate the selectivity of the newly developed compounds against cancer cells with respect to non-cancer ones. To this attempt, compounds were screened against a human non-transformed, highly replicating cell line, the human embryonic kidney 293 (HEK293) cells. Remarkably, compounds belonging to both the pyrrolo[1,2-*h*][1,7]naphthyridinone and pyrido[2,3-*c*]pyrrolo[1,2-*a*]azepinone series were completely ineffective against non-cancer cells, thus attesting their preferential activity against cancer cells (Table S1).

### Cell-free and in cells photogeneration of ROS

To corroborate the above reported hypothesis and to confirm the ability of all derivatives to form ROS when UVA irradiated, experiments in both cell-free and MDA-MB-231 human TNBC cells were performed. Concerning cell-free experiments, two in solution assays for singlet oxygen and superoxide anion have demonstrated to be highly predictable for many photosensitizing molecules (Kraljic and El Mohsni 1978; Pathak and Joshi 1984; Dalla Via et al. 2015; Zhang et al. 2021).

#### Cell-free ROS generation

With the two assays shown below, it is possible to distinguish the capacity of the test compounds of producing singlet oxygen over superoxide anion and therefore predict their behaviour as photosensitizers.

**Fig. 2:**
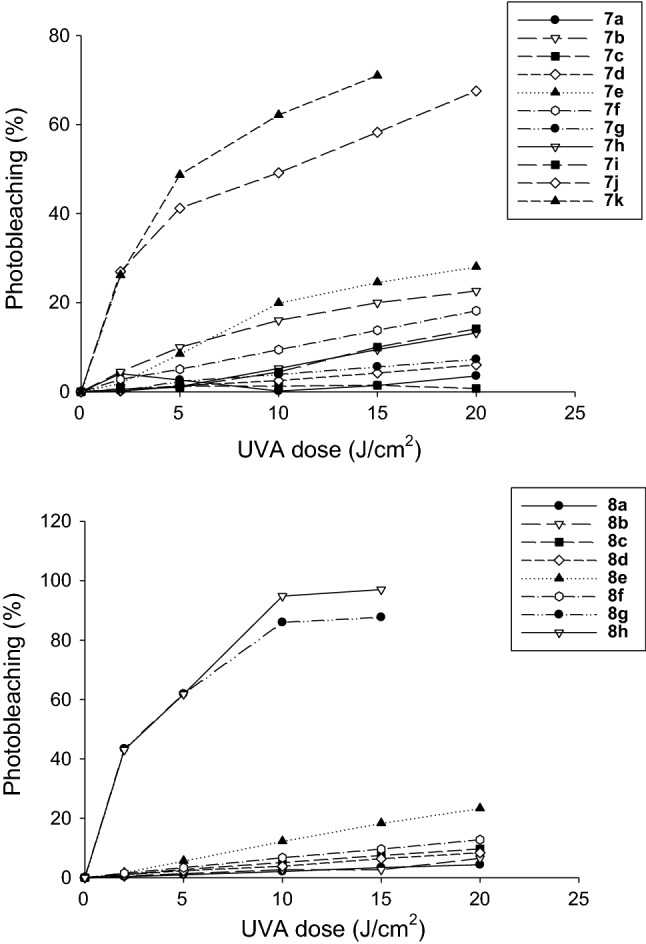
Variation of RNO absorbance at 440 nm as indication of singlet oxygen production by tested compounds under increasing UVA light doses (0–20 J/cm^2^)

The percentage of RNO photobleaching of tested compounds under increasing light doses, which is a measure of singlet oxygen generation, is shown in Fig. 2. The majority of the compounds, with the exception of **7a** and **7c**, were able to generate singlet oxygen in a light dose-dependent manner (0–20 J/cm^2^), thus attesting that a higher UVA dose increased the singlet oxygen concentration by these compounds and, as a consequence, led to stronger oxidation of RNO/higher percentage of photobleaching. Among the two series, derivatives **7j** and **7k** as well as **8g** and **8h** were the ones with a higher percentage of RNO photobleaching, thus indicating their greater ability of singlet oxygen generation.

**Fig. 3:**
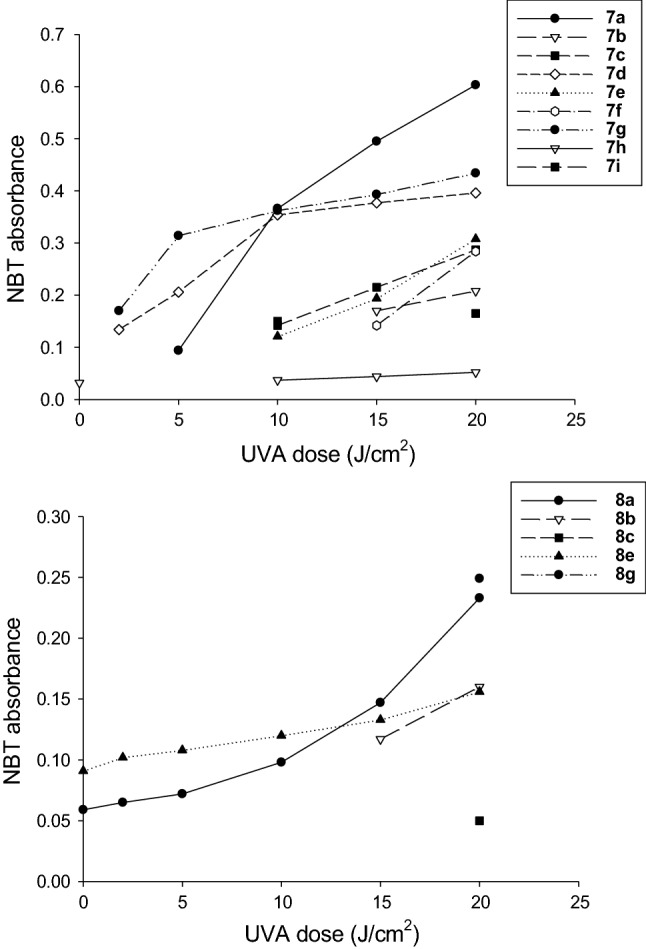
Variation of NBT at 560 nm as indication of superoxide anion production by tested compounds under increasing UVA light doses (0–20 J/cm^2^)

Additionally, in Fig. 3 the changes in the absorbance values of NBT at 560 nm under increasing light doses are depicted. Some of the compounds only registered the production of superoxide anion under specific light dose(s) rather than under the whole light dose range. Compounds **7j**, **7k**, **8d**, **8f**, and **8h** production was not reported since they did not produce superoxide anion at any light dose. Likely, superoxide anion is a free radical with a short half-life, making its measurement more difficult than singlet oxygen especially when produced in very low amount. Among all, the most effective in generating a high concentration of superoxide anion was compound **7a**. Moreover, from a biological point of view, this radical species despite its high reduction potential of + 0.94 V, can oxidize very few biological compounds (D’Autréaux and Toledano 2007; Krumova et al. 2016), thus O_2_˙^−^ is less effective in destroying cell components than singlet oxygen and therefore less powerful in provoking a general toxicity against treated cancer cells (see cell viability experiments).

#### Intracellular ROS generation

In order to understand if the intracellular action mechanism of our compounds was dependent on ROS production in intact cancer cells, the most representative compounds **7h** and **8e** were evaluated for their ability to increase intracellular basal ROS production in TNBC cells. With this kind of assay, all reactive oxygen species are detected without distinction among singlet oxygen, superoxide, hypochlorite, nitric oxide and hydrogen peroxide.

**Fig. 4:**
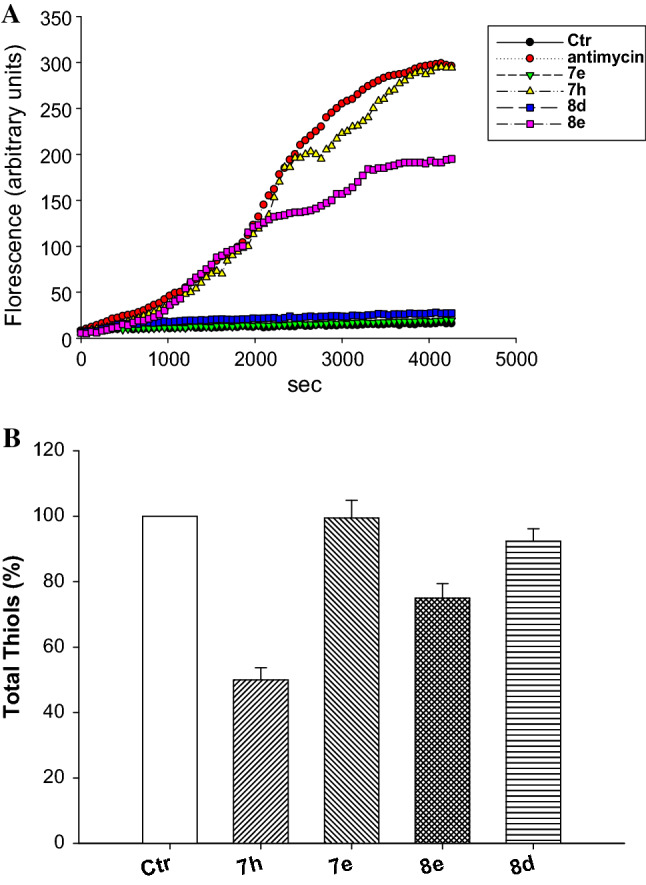
Cellular ROS production (**A**) and total thiol redox state (**B**). **A** Effect of UVA activated tested compounds (25 µM) or antimycin (3 µM) on hydrogen peroxide formation in MDA-MB-231 cancer cells. **B** Sulfhydryl content in MDA-MB-231 cancer cells incubated for 24 h with UVA activated tested compounds. The sulfhydryl group amount was determined by the DTNB assay. Error bars indicate S.D

ROS production under light (UVA, 2.0 J/cm^2^) was evaluated in MDA-MB-231 cell samples treated with increasing concentrations of tested compounds or antimycin, a potent inhibitor of Complex III of mitochondria electron chain, used as a positive control under the same experimental conditions (Fig. 4).

Treatment of MDA-MB-231 cells with **7h** and **8e** determined the highest increase in cellular ROS production, in a time-dependent manner (Fig. 4A). Remarkably, cells treated with **7h** showed a hydrogen peroxide content comparable to that induced by antimycin. Conversely, compounds **7e** and **8d** were hardly effective in inducing any ROS basal level production increase.

Coherently, intracellular thiols were significantly decreased by treatment with **7h** and **8e** in a time-dependent manner. Interestingly, **7h** was much more effective than **8e**, being able to decrease intracellular sulfhydryl content of about 45% after 24 h of exposure (Fig. 4B).

### Mitochondrial membrane potential loss and apoptotic cell death

Induction of ROS production can in turn prompt the collapse of mitochondrial membrane potential as well as loss of mitochondrial integrity and functioning. We hence evaluated the effect determined by treatment with representative compounds in terms of mitochondrial membrane potential changes. MDA-MB-231 TNBC cells were treated with tested compounds upon UVA irradiation and the percentage of cells with hypopolarized mitochondrial membrane potential was determined fluorometrically by means of the Mito-ID® Membrane Potential Kit.

**Fig. 5:**
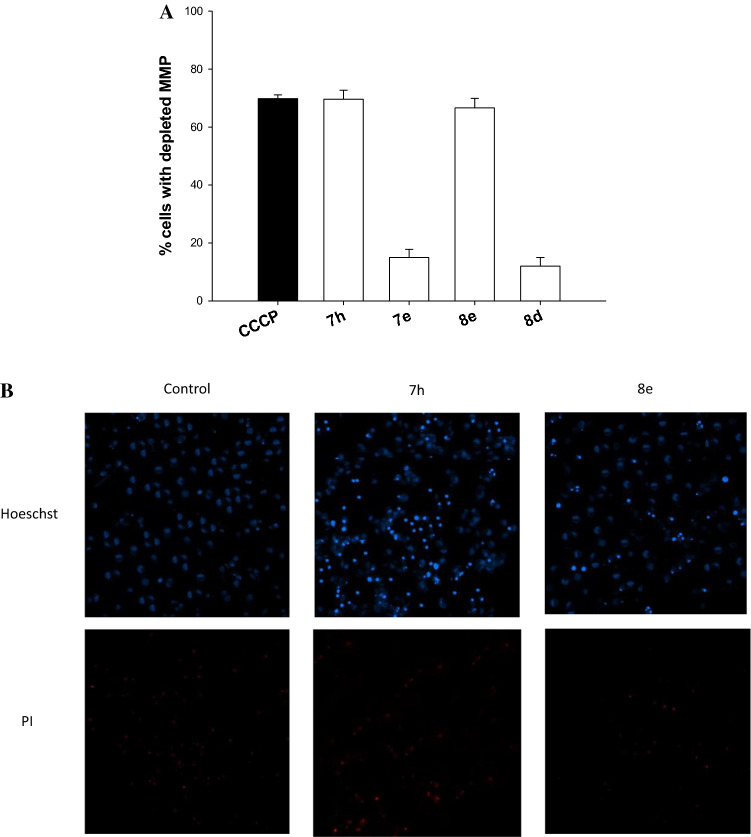
Effects on mitochondrial membrane potential (**A**) and cell death induction (**B**) in human breast MDA-MB-231 cancer cells. **A** Cells were treated with **7h** and **8e** and stained with TMRM (10 nM). Fluorescence was estimated at 490 nm (excitation) and 590 nm (emission). Data are the means of five independent experiments. Error bars indicate S.D. * P < 0.05. **B** Cells were treated with concentrations of **7h** and **8e** equal to IC_50_ for 24 h and stained with the fluorescent dyes Hoechst 33342 and PI

As summarized in Fig. 5A, a significant increase of cells with depolarized mitochondria was observed after treatment with compounds **7h** and **8e** which was like that exerted by the positive control CCCP (Carbonyl cyanide 3-chlorophenyl-hydrazone). On the contrary, treatment with **7e** and **8d** did not induce a substantial change in mitochondrial membrane potential of TNBC cells.

Loss of mitochondrial transmembrane potential is a crucial step in the activation of intrinsic apoptotic cell death. Hence, the ability to induce apoptosis by representative compounds **7h** and **8e** was assessed in human TNBC cells. MDA-MB-231 cells were treated with IC_50_ doses of tested compounds and apoptotic cell death induction was confirmed through a Hoechst 33342 staining assay.

As reported in Fig. 5B, cells treated with **7h** and **8e**, as compared with control untreated cells, were positive to Hoechst 33342 staining, and displayed chromatin condensation and fragmentation as well as pyknotic nuclei, typical features of apoptosis. On the other hand, treated cells were poor stained with PI, thus indicating the absence of necrosis induction.

### Photostability experiments

**Fig. 6:**
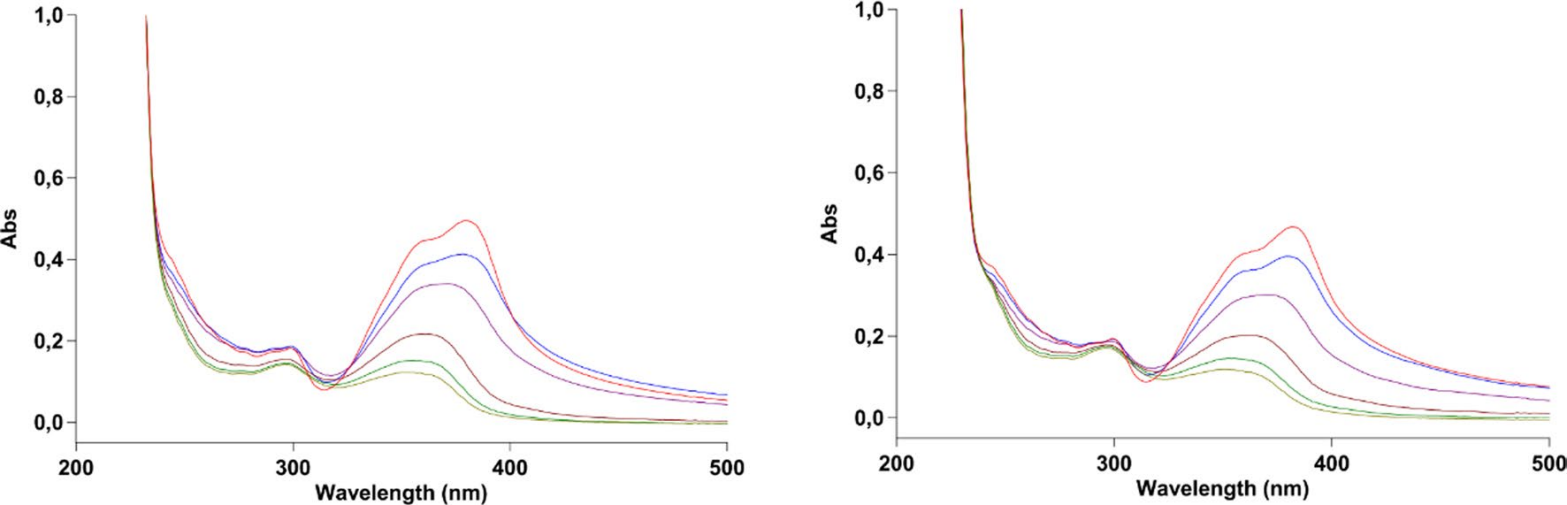
UV–Vis Absorption spectra of **7h** (left) and **8e** (right) in PBS irradiated under 0, 2, 5, 10, 15 and 20 J/cm^2^ with UVA light. 0 J/cm^2^, red spectrum; 20 J/cm^2^, light green spectrum

The most significant **7h** and **8e** pyridone derivatives were tested for their stability under UVA irradiation. These data might be useful to understand the mechanism of action of the compounds apart from the production of ROS. As shown by the changes of their UV–Vis spectra under increasing UVA doses (Fig. 6), compound taken in the dark = 0 J/cm^2^ = red spectrum; compound under 20 J/cm^2^ of UVA light = light green spectrum), the tested compounds were very unstable under light and behaved very similarly. Indeed, their main absorption peak gradually decreased under irradiation accompanied by a blue shift of the maximum and the change of its shape, suggesting the loss of the original structure. Moreover, isosbestic points were visible, indicating the formation of one or more new stable species in equilibrium with the intact dark compound.

In addition, to test the role of the photodegradation products on the photocytotoxicity profiles of the most representative derivatives **7h** and **8e**, the cytotoxic activity of the photodegraded mixture was evaluated by treating TNBC cells with the corresponding pre-irradiated (20 J/cm^2^) PBS solutions. No cytotoxic activity was evidenced (IC_50_ over 50 µM) clearly attesting that the photodegradation products did not frankly contribute to the cytotoxic activity of the test compounds; rather, these compounds lose their activity when photodegraded and suggest that they mainly act by inducing intracellular ROS.

By roughly calculating the decrease of absorbance of the main UV–Vis peaks (data non shown), the photodegradation was very weak under the dose of light used during the cytotoxic experiments (2 J/cm^2^, blue line in Fig. 5) and the yield of photoproducts formed able to participate to the activity of **7h** and **8e** was most likely not significant for their contribution in their general cytotoxicity. Anyway, even in very high amount (after 20 J/cm^2^ of UVA, light green line in Fig. 5), they were not active at all as discussed above.

### Cellular accumulation and biodistribution studies

With the aim of correlating cellular phototoxic activity with intracellular accumulation of tested compounds, we performed some preliminary in cell biodistribution studies. By exploiting their fluorescence characteristics, the most effective compounds **7h** and **8e**, as well as a completely ineffective derivative, namely **8h**, were evaluated for their ability to enter MDA-MB-231 TNBC cells and localize into different cellular compartments by confocal microscopy analysis.

**Fig. 7:**
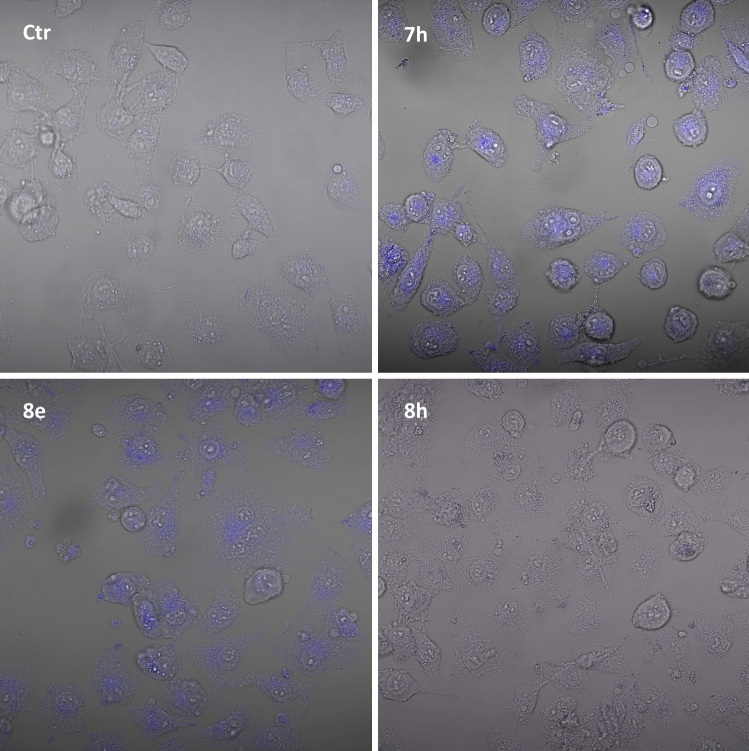
Intracellular accumulation studies. MDA-MB-231 TNBC cells were treated with **7h, 8e** and **8h** upon irradiation and live samples were examined at × 20 magnification by a confocal microscope

As evident form micrographs reported in Fig. 7, the most photocytotoxic compounds **7h** and **8e** were able to significantly enter cancer cell membrane and accumulate in cytoplasm. Compounds preferentially localize in the perinuclear space, with minimal accumulation in the periplasmatic site. Conversely, compound **8h** was barely effective in accumulating into cancer cells, and only low levels of fluorescence were detected in the cytoplasm of treated cancer cells.

Overall, these cellular accumulation studies clearly support the hypothesis that the cell killing inactivity of compound **8h** can be explained by taking into consideration its scarce efficacy to permeate TNBC cells. Despite behaving as PS in cell-free experiments, **8h** was found completely ineffective in inhibiting cancer cell proliferation upon irradiation.

## Discussion

Photochemotherapy broadly refers to a treatment method that uses a photosensitizer (PS) activated by a selected light irradiation. The combination of both produces a better effect than the use of one of these treatments alone (Ledo and Ledo 2000). This therapeutic technique comprises PUVA (psoralen-UV-A) therapy, in which a psoralen derivative damages DNA of proliferating cells under the exposure to ultraviolet A (UV-A) radiation (Schneider et al. 2008) and photodynamic therapy (PDT) that relies on the red-light activation of a porphyrin-like derivative that generates reactive oxygen species leading to the destruction of unwanted cells and diseased tissues (Benov 2015). The lack of toxicity of the above photosensitizers in the dark and the selective illumination of the targeted area avoids the general toxicity that is one of the main drawbacks of the conventional chemotherapy against cancer. Therefore, the ideal PS would be a stable drug, with specific uptake by the targeted tissues, minimal dark cytotoxicity, strong absorption at relatively long wavelengths (600–800 nm) at which the light can penetrate efficiently into the tissues, rapid clearance to avoid phototoxic side effects, being amphiphilic, chemically pure and easy to access or synthesize (Dias et al. 2021). In the case of PS activated by less penetrating shorter wavelengths, such as psoralen derivatives activated by UVA light and, as demonstrated more recently, even by blue light (Sturaro et al. 2021), their application against proliferating cells contemplates the treatment of skin diseases, i.e. psoriasis and neurodermitis, or an extracorporeal treatment, the so called extracorporeal photopheresis (ECP) against some immunological disorders, i.e., cutaneous T cell lymphoma and graft versus host disease (Drexler et al. 2020). Although a big progress has been done towards the understanding of TNBC’s biology and the development of more efficient treatment regimens, drug resistance, strong side effects and episodes of recurrence are still common among patients with this aggressive disease. Intensive research has been conducted to uncover a new therapeutical approach which maintains a patient’s quality of life and has a more localized action. Quinazoline derivatives arose from this diligent search as a promising direction in the treatment of this life- threatening cancer disease. Their enormous potential is the motivation for this study, whose aims consisted of testing the photocytotoxic activity of nineteen newly synthesised photosensitizing pyridone derivatives on a TNBC cancer cell line (MDA-MB-231) under UV-A light and study their ability to produce ROS as the main mechanism of their action.

The demonstrated antiproliferative activity in the micromolar range of some of these compounds in combination with a small non-toxic dose of UVA light (2.0 J/cm^2^), their inactivity in the dark against both normal and cancer cells, their apoptotic mechanism of cell death make some of these compounds as a very promising alternative to the conventional and often not effective therapies against TNBC. Interestingly, all the compounds presenting a bromine as substituent at the pentatomic ring were able to generate high yields of singlet oxygen. This is in line with the effect of the heavy atom in the molecular structure which increases the spin orbit coupling between singlet and triplet states, leading to more efficient ISC with respect to the non-halogenated compounds (Lai et al. 2021). Unfortunately, these compounds bearing the heavy halogen bromine atom as inducer of singlet oxygen production could not enter the cells so efficiently and their potential higher activity with respect the other compounds was lost in the presence of cancer cells. Indeed, the localization of the compounds inside the cells confirmed this assumption.

It must be noted that, as in the case of PUVA, we evaluated the toxicity of these compounds under UVA light, where all absorbed significantly; but unfortunately, UVA light penetrates the tissues much less than the red light used in PDT. Theoretically, PUVA therapy could not be used to treat solid internal cancers or deep skin cancers as PDT. However, a direct irradiation of the diseased area in the presence of our compounds to get a clean tissue during and after the surgical removal of the tumour could be suggested. Moreover, for superficial breast cancers the use of a catheter for the instillation of the photoactive principle followed by irradiation through a probe, equipped with a tip emitting UVA light, could be another option for these potential phototherapeutic compounds.

## Supplementary Information

Below is the link to the electronic supplementary material.

Supplementary file1 (DOCX 1996 kb)

## References

[CR1] Ali S, Buczek D, Jassem J (2020). Changing paradigms in breast cancer treatment. Eur J Transl Clin Med.

[CR2] Barraja P, Diana P, Montalbano A, Dattolo G, Cirrincione G, Viola G, Vedaldi D, Dall'Acqua F (2006). Pyrrolo[2,3-h]quinolinones: a new ring system with potent photoantiproliferative activity. Bioorg Med Chem.

[CR3] Barraja P, Caracausi L, Diana P, Carbone A, Montalbano A, Cirrincione G, Brun P, Castagliuolo I, Dall'Acqua F, Vedaldi D, Salvador A (2010). Synthesis of pyrrolo[3,2-h]quinolinones with good photochemotherapeutic activity and no DNA damage. Bioorg Med Chem.

[CR4] Barraja P, Diana P, Montalbano A, Carbone A, Viola G, Basso G, Salvador A, Vedaldi D, Dall'Acqua F, Cirrincione G (2011). Pyrrolo[3,4-h]quinolinones a new class of photochemotherapeutic agents. Bioorg Med Chem.

[CR5] Barreca M, Ingarra AM, Raimondi MV, Spanò V, De Franco M, Menilli L, Gandin V, Miolo G, Barraja P, Montalbano A (2022). Insight on pyrimido[5,4-g]indolizine and pyrimido[4,5-c]pyrrolo[1,2-a]azepine systems as promising photosensitizers on malignant cells. Eur J Med Chem.

[CR6] Barreca M, Spanò V, Raimondi MV, Bivacqua R, Giuffrida S, Montalbano A, Cavalli A, Bertoni F, Barraja P (2022). GPCR inhibition in treating lymphoma. ACS Med Chem Lett.

[CR7] Benov L (2015). Photodynamic therapy: current status and future directions. Med Princ Pract.

[CR8] Bergin ART, Loi S (2019) Triple-negative breast cancer: recent treatment advances [version 1; peer review: 2 approved]. F1000Research 8:1342. 10.12688/f1000research.18888.1 10.12688/f1000research.18888.1 PMC6681627 31448088

[CR9] Cilibrasi V, Spanò V, Bortolozzi R, Barreca M, Raimondi MV, Maruca A, Montalbano A, Alcaro S, Ronca R, Viola G, Barraja P (2022). Synthesis of 2H-Imidazo[2′,1’:2,3] [1,3]thiazolo[4,5-e]isoindol-8-yl-phenylureas with promising therapeutic features for the treatment of acute myeloid leukemia (AML) with FLT3/ITD mutations. Eur J Med Chem.

[CR10] Criscitiello C, Azim HA, Schouten PC, Linn SC, Sotiriou C (2012). Understanding the biology of triple-negative breast cancer. Ann Oncol.

[CR11] D’Autréaux B, Toledano MB (2007). ROS as signalling molecules: mechanisms that generate specificity in ROS homeostasis. Nat Rev Mol Cell Biol.

[CR12] Dalla Via L, Marzaro G, Mazzoli A, Chilin A, Miolo G (2015). Photobiological properties of 3-psoralenacetic acids. Photochem Photobiol Sci.

[CR13] Dias CJ, Helguero L, Faustino MAF (2021). Current photoactive molecules for targeted therapy of triple-negative breast cancer. Molecules.

[CR14] Drexler B, Buser A, Infanti L, Stehle G, Halter J, Holbro A (2020). Extracorporeal photopheresis in Graft-versus-host disease. Transfus Med Hemother.

[CR15] Kraljic I, El Mohsni S (1978). A new method for the detection of singlet oxygen in acqueosus solutions. Photochem Photobiol.

[CR16] Krumova K, Cosa G, Aubry J, Kanofsky JR (2016) Section I: Fundamentals. In: Singlet oxygen : applications in biosciences and nanosciences, pp 1–2. 10.1039/9781782622208-00001

[CR17] Labbozzetta M, Barreca M, Spanò V, Raimondi MV, Poma P, Notarbartolo M, Barraja P, Montalbano A (2022). Novel insights on [1,2]oxazolo[5,4-e]isoindoles on multidrug resistant acute myeloid leukemia cell line. Drug Dev Res.

[CR18] Lai L, Fang B, Fan M, Cheng W, Meizhen Y (2021). Modulating room-temperature phosphorescence through the synergistic effect of heavy-atom effect and halogen bonding. J Phys Chem C.

[CR19] Ledo E, Ledo A (2000). Phototherapy, photochemotherapy, and photodynamic therapy: unapproved uses or indications. Clin Dermatol.

[CR20] Liu J, Ming B, Gong GH, Wang D, Bao GL, Yu LJ (2018). Current research on anti-breast cancer synthetic compounds. RSC Adv.

[CR21] Lüönd F, Tiede S, Christofori G (2021). Breast cancer as an example of tumour heterogeneity and tumour cell plasticity during malignant progression. Br J Cancer.

[CR22] Miolo G, Salvador A, Mazzoli A, Spallett A, Marzaro G, Chilin A (2014). Photochemical and photobiological studies on furoquinazolines as new psoralen analogs. J Photochem Photobiol B.

[CR23] Pathak MA, Joshi PC (1984). Production of active oxygen species (1O2 and O2⨪) by psoralens and ultraviolet radiation (320–400 nm). BBA.

[CR24] Rigobello MP, Gandin V, Folda A, Rundlof AK, Fernandes AP, Bindoli A, Marzano C, Bjornstedt M (2009). Treatment of human cancer cells with selenite or tellurite in combination with auranofin enhances cell death due to redox shift. Free Radic Biol Med.

[CR25] Schneider LA, Hinrichs R, Scharffetter-Kochanek K (2008). Phototherapy and photochemotherapy. Clin Dermatol.

[CR26] Spanò V, Giallombardo D, Cilibrasi V, Parrino B, Carbone A, Montalbano A, Frasson I, Salvador A, Richter SN, Doria F, Freccero M, Cascioferro S, Diana P, Cirrincione G, Barraja P (2017). Pyrrolo[3′,2′:6,7]cyclohepta[1,2-b]pyridines with potent photo-antiproliferative activity. Eur J Med Chem.

[CR27] Spanò V, Barreca M, Cilibrasi V, Genovese M, Renda M, Montalbano A, Galietta LJV, Barraja P (2021). Evaluation of fused pyrrolothiazole systems as correctors of mutant CFTR protein. Molecules.

[CR28] Spanò V, Barreca M, Rocca R, Bortolozzi R, Bai R, Carbone A, Raimondi MV, Palumbo Piccionello A, Montalbano A, Alcaro S, Hamel E, Viola G, Barraja P (2021). Insight on [1,3]thiazolo[4,5-e]isoindoles as tubulin polymerization inhibitors. Eur J Med Chem.

[CR29] Sporikova Z, Koudelakova V, Trojanec R, Hajduch M (2018). Genetic markers in triple-negative breast cancer. Clin Breast Cancer.

[CR30] Sturaro G, Tasso A, Menilli L, Di Liddo R, Miolo G, Conconi MT (2021). 4,6,4′-trimethylangelicin photoactivated by blue light might represent an interesting option for photochemotherapy of non-invasive bladder carcinoma: an in vitro study on T24 cells. Biomolecules.

[CR31] Tong CWS, Wu M, Cho WCS, To KKW (2018). Recent advances in the treatment of breast cancer. Front Oncol.

[CR32] Yao H, He G, Yan S, Chen C, Song L, Rosol TJ, Deng X (2017). Triple-negative breast cancer: is there a treatment on the horizon?. Oncotarget.

[CR33] Zhang Z, Fan J, Du J, Peng X (2021). Two-channel responsive luminescent chemosensors for dioxygen species: Molecular oxygen, singlet oxygen and superoxide anion. Coord Chem Rev.

