# Supplementary material for: New tricyclic systems as photosensitizers towards triple negative breast cancer cells
Source: Arch Pharm Res. 2022 Nov 18;45(11):806–21. doi: 10.1007/s12272-022-01414-1 (PMC9701179; doi:10.1007/s12272-022-01414-1)
Supplement: Supplementary file 1 — Supplementary file1 (DOCX 1996 kb) [file 12272_2022_1414_MOESM1_ESM.docx]

**New tricyclic systems as photosensitizers towards triple negative breast cancer cells**

Marilia Barreca^1^, Angela Maria Ingarra^1^, Maria Valeria Raimondi^1^, Virginia Spanò^1,*^, Antonio Palumbo Piccionello^1^, Michele De Franco^2^, Luca Menilli^2^, Valentina Gandin^2^, Giorgia Miolo^2,*^, Paola Barraja^1^, Alessandra Montalbano^1^

^1^Department of Biological, Chemical, and Pharmaceutical Sciences and Technologies (STEBICEF), University of Palermo, Via Archirafi 32, 90123 Palermo, Italy

^2^Department of Pharmaceutical and Pharmacological Sciences, University of Padova, Via Marzolo 5, 35131 Padova, Italy

^*^**Corresponding author**: [virginia.spano@unipa.it](mailto:virginia.spano@unipa.it), [giorgia.miolo@unipd.it](javascript:void(window.open('/imp/dynamic.php?page=compose&to=giorgia.miolo%40unipd.it&popup=1%27,%27%27,%27width=820,height=610,status=1,scrollbars=yes,resizable=yes%27)))

**Table of contents:**

1. **Table S1. Cytotoxicity against human embryonic kidney 293 (HEK293) cells**
2. **Figure S1.** ^1^H and ^13^C NMR of compound **7a**
3. **Figure S2.** ^1^H and ^13^C NMR of compound **7b**
4. **Figure S3.** ^1^H and ^13^C NMR of compound **7c**
5. **Figure S4.** ^1^H and ^13^C NMR of compound **7d**
6. **Figure S5.** ^1^H and ^13^C NMR of compound **7e**
7. **Figure S6.** ^1^H and ^13^C NMR of compound **7f**
8. **Figure S7.** ^1^H and ^13^C NMR of compound **7g**
9. **Figure S8.** ^1^H and ^13^C NMR of compound **7h**
10. **Figure S9.** ^1^H and ^13^C NMR of compound **7i**
11. **Figure S10.** ^1^H and ^13^C NMR of compound **7j**
12. **Figure S11.** ^1^H and ^13^C NMR of compound **7k**
13. **Figure S12.** ^1^H and ^13^C NMR of compound **8a**
14. **Figure S13.** ^1^H and ^13^C NMR of compound **8b**
15. **Figure S14.** ^1^H and ^13^C NMR of compound **8c**
16. **Figure S15.** ^1^H and ^13^C NMR of compound **8d**
17. **Figure S16.** ^1^H and ^13^C NMR of compound **8e**
18. **Figure S17.** ^1^H and ^13^C NMR of compound **8f**
19. **Figure S18.** ^1^H and ^13^C NMR of compound **8g**
20. **Figure S19.** ^1^H and ^13^C NMR of compound **8h**

**Table S1.** Cytotoxicity against human embryonic kidney 293 (HEK293) cells.

Cells (5×10^3^ mL^-1^) were treated for 1 h with increasing concentrations of tested compounds and following the medium was replaced fresh one. Cells were recovered for a total of 24 h. The cytotoxicity was assessed by the MTT test. IC_50_ values were calculated by a four-parameter logistic model 4-PL (P<0.05). S.D. = standard deviation. ND = not detected.

**Figure S1.** ^1^H and ^13^C NMR of compound **7a**

**
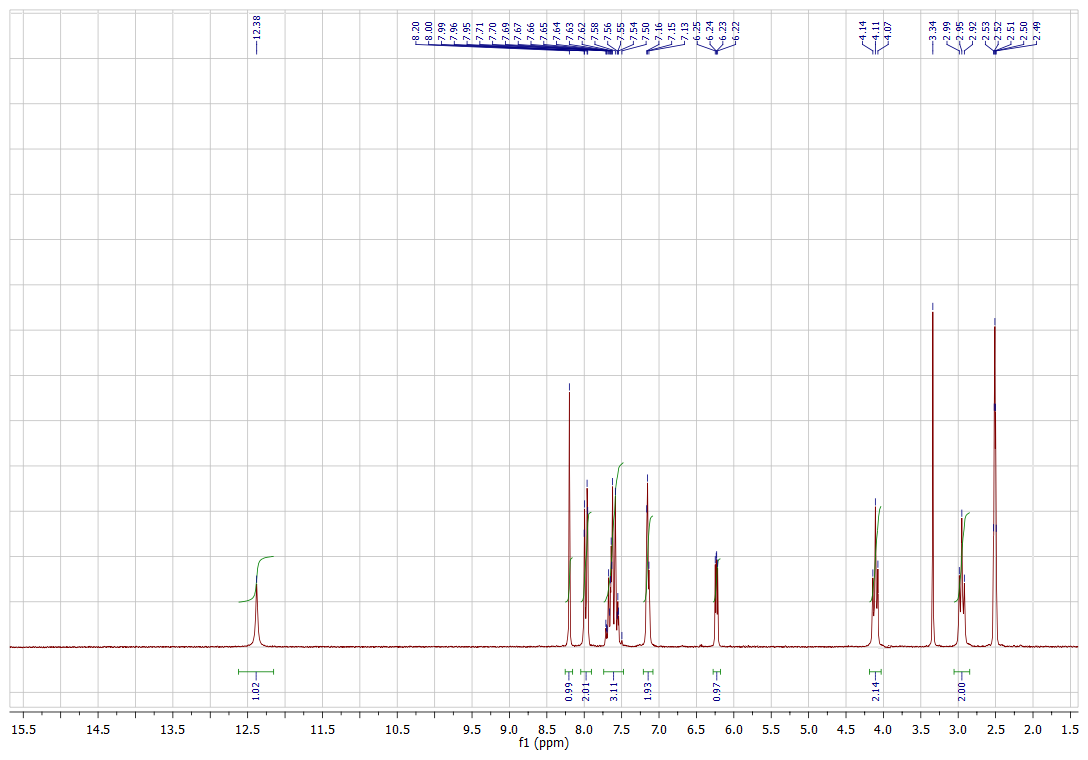
**

**
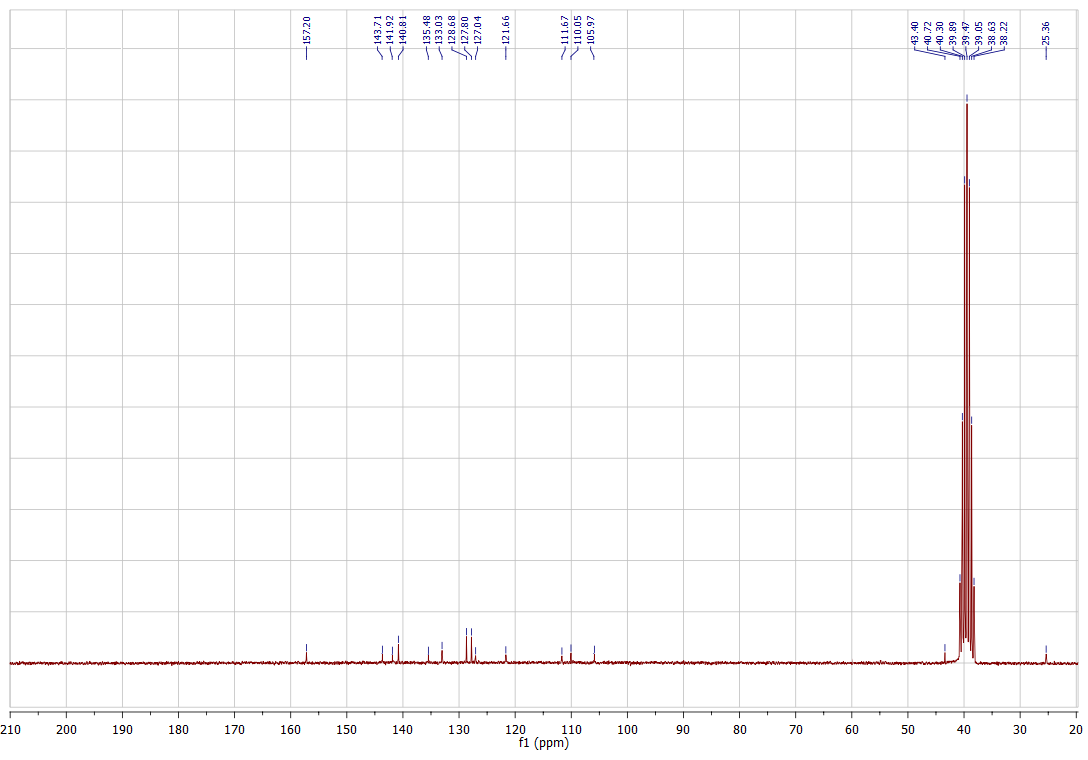
**

**Figure S2.** ^1^H and ^13^C NMR of compound **7b**

**
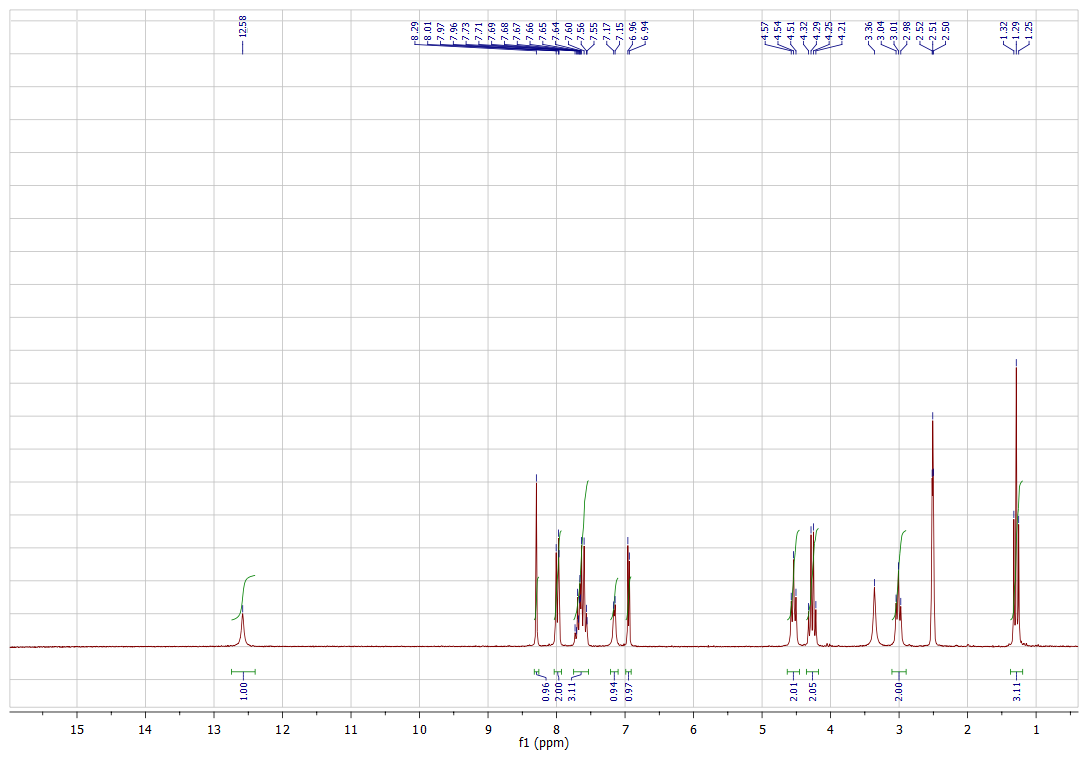
**

**
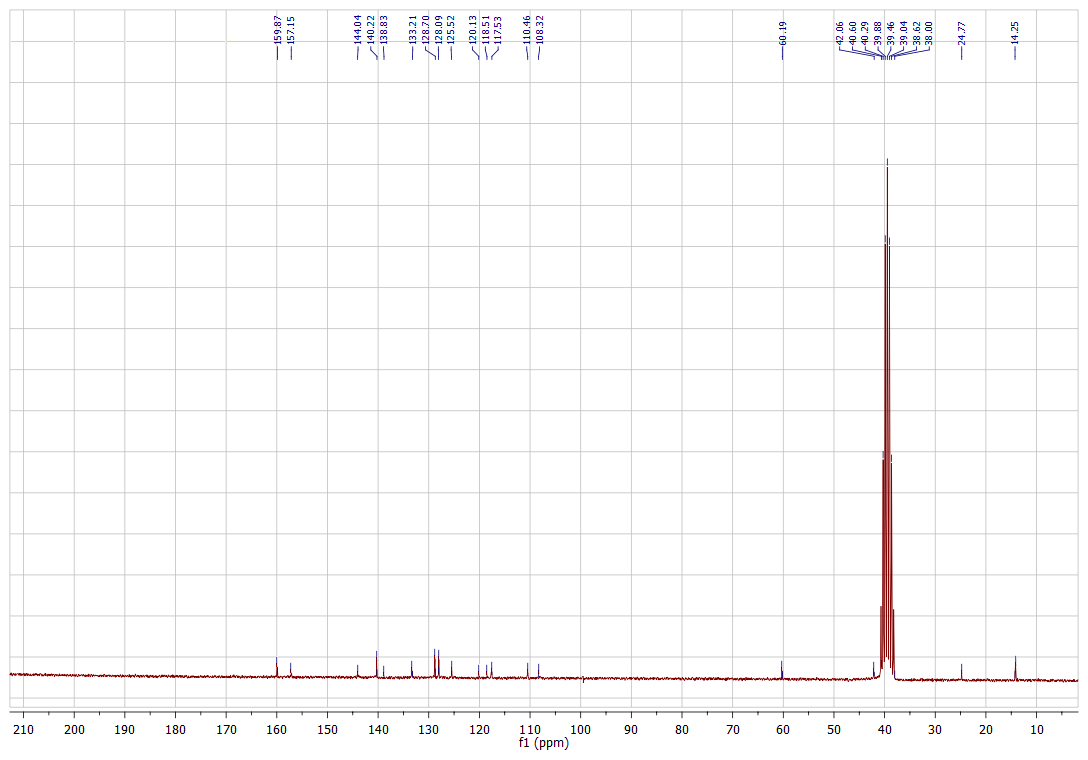
**

**Figure S3.** ^1^H and ^13^C NMR of compound **7c**

**
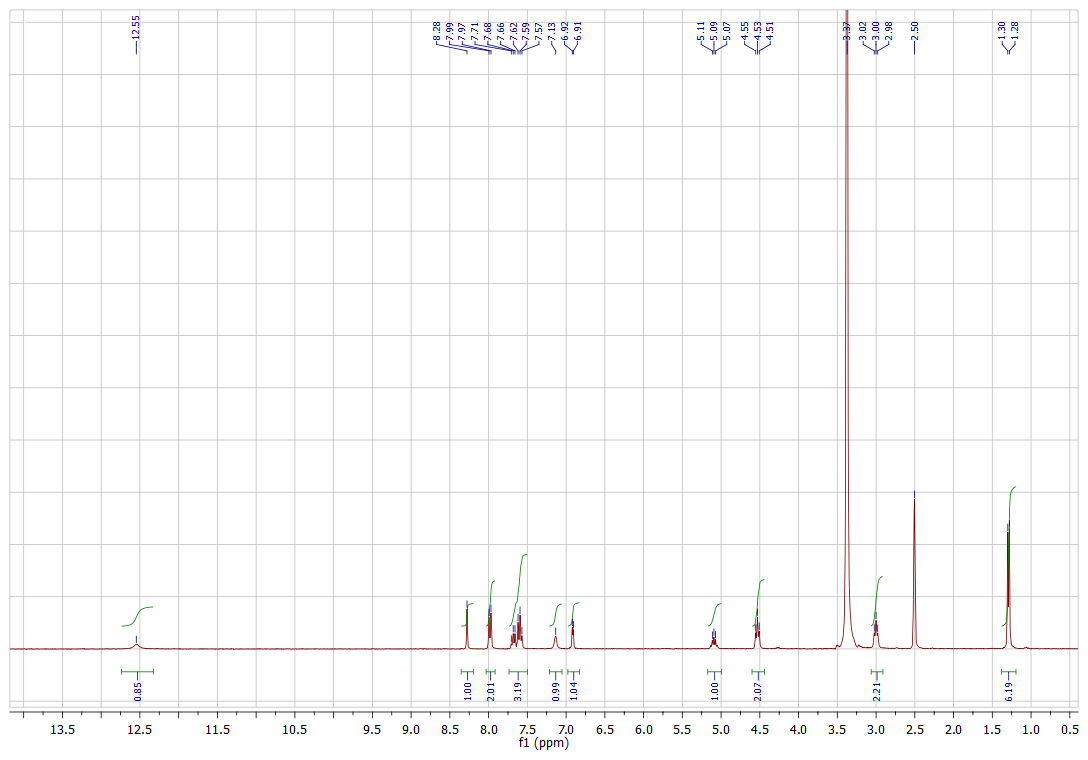
**

**
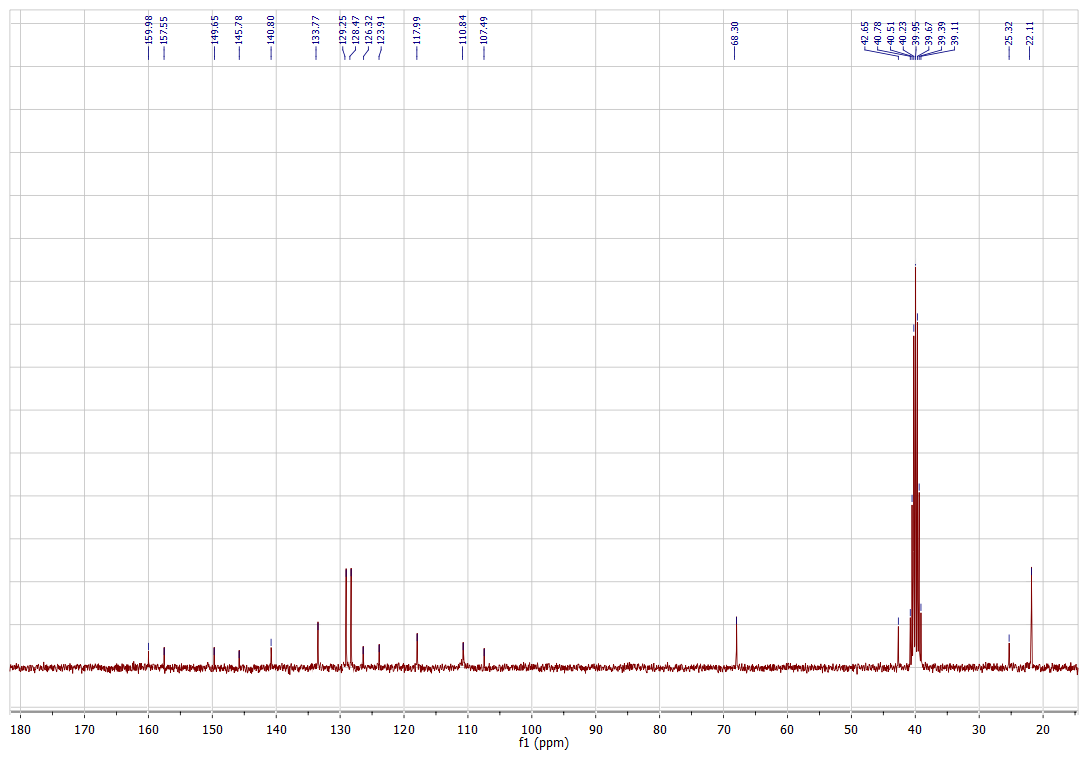
**

**Figure S4.** ^1^H and ^13^C NMR of compound **7d**

**
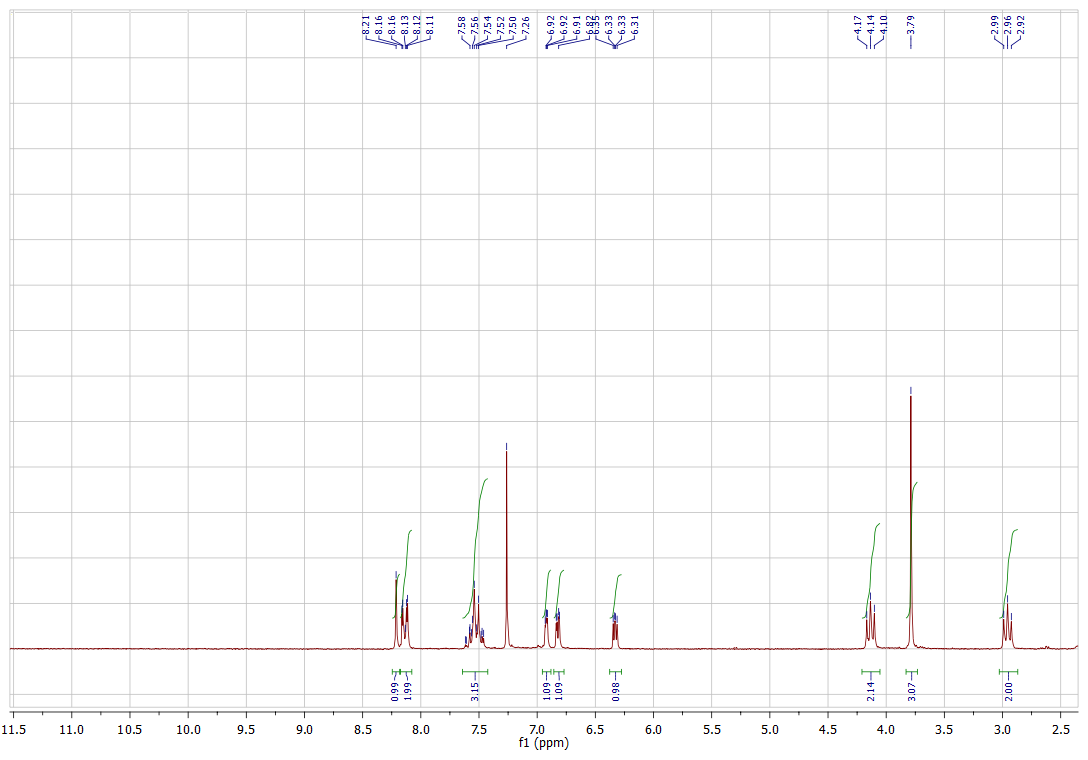
**

**
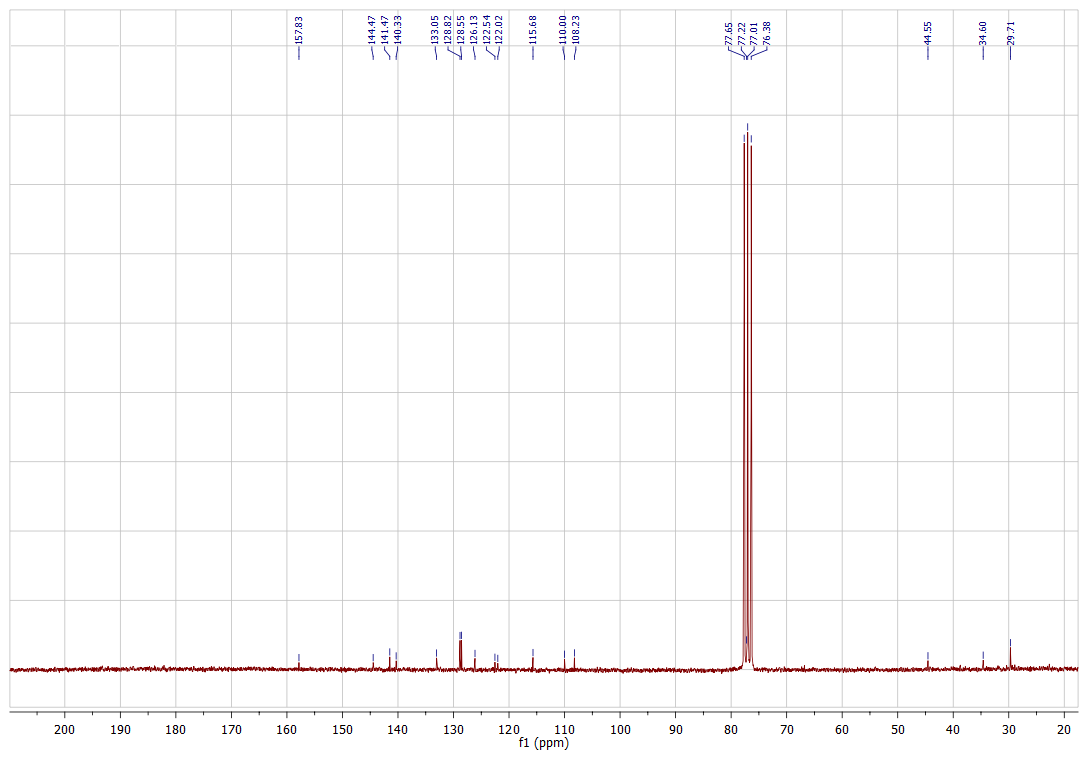
**

**Figure S5.** ^1^H and ^13^C NMR of compound **7e**

**
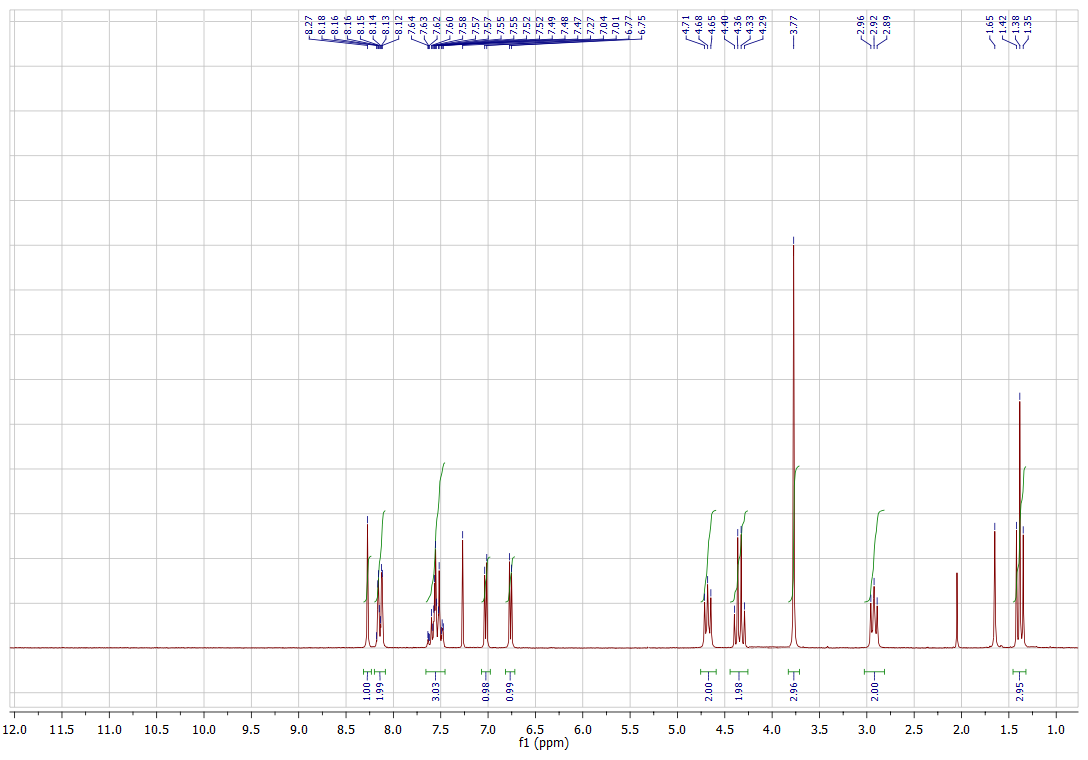
**

**
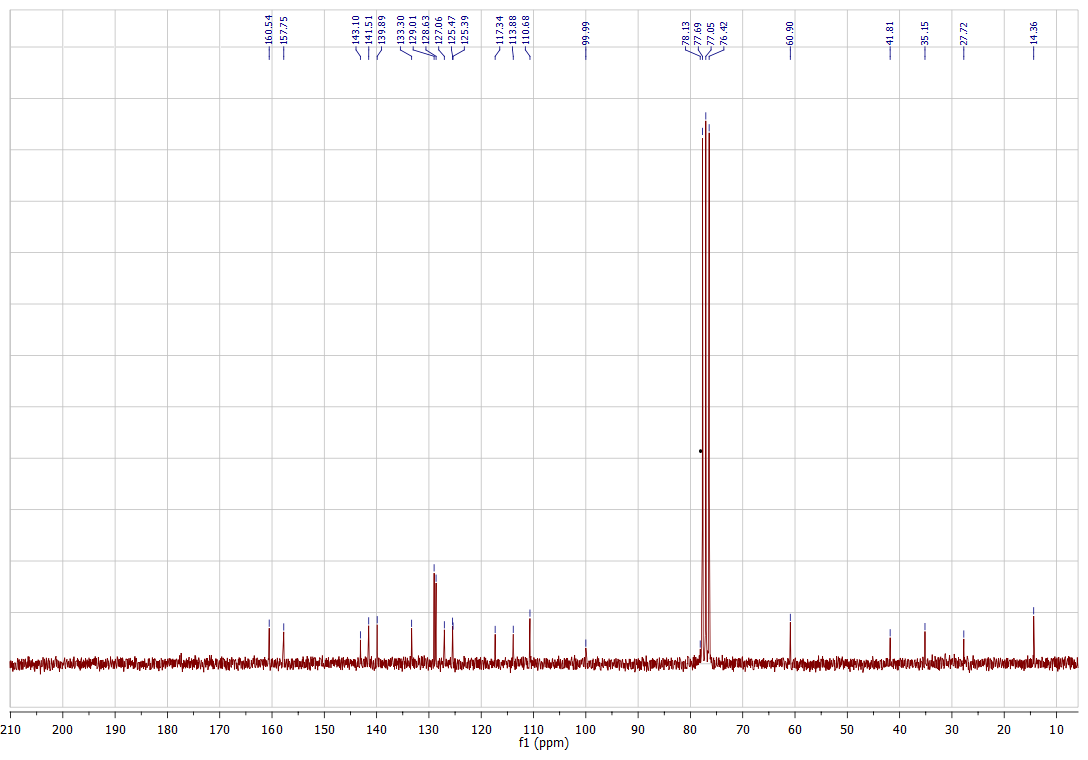
**

**Figure S6.** ^1^H and ^13^C NMR of compound **7f**

**
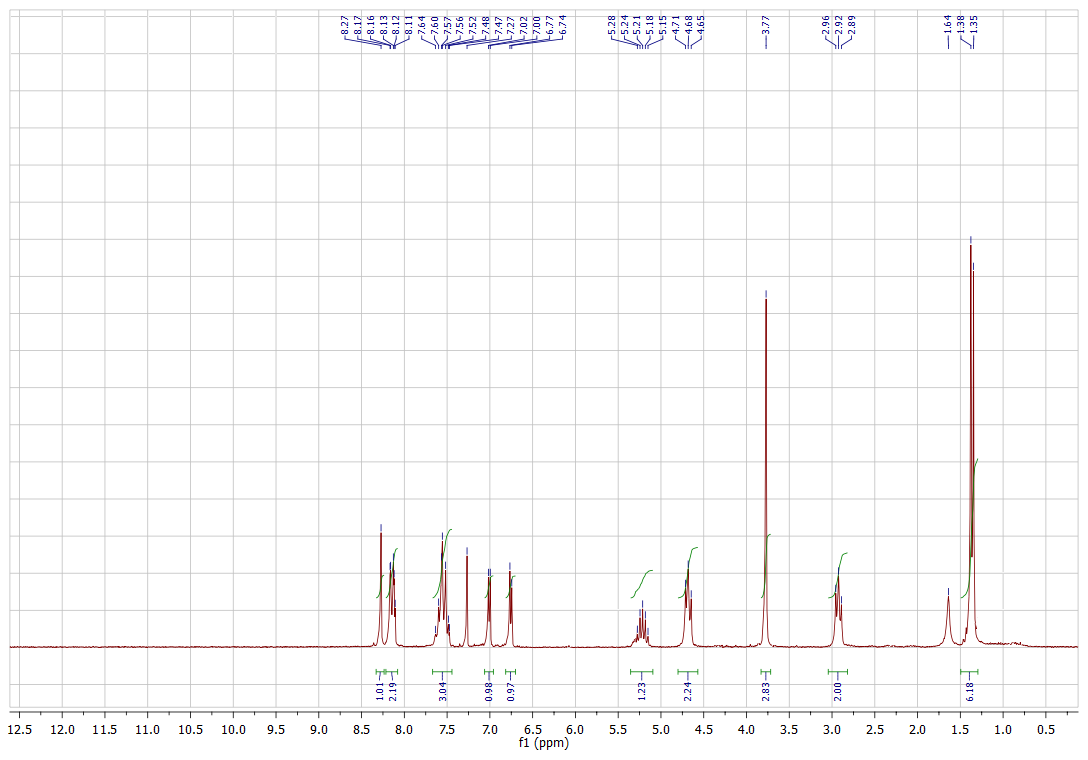
**

**
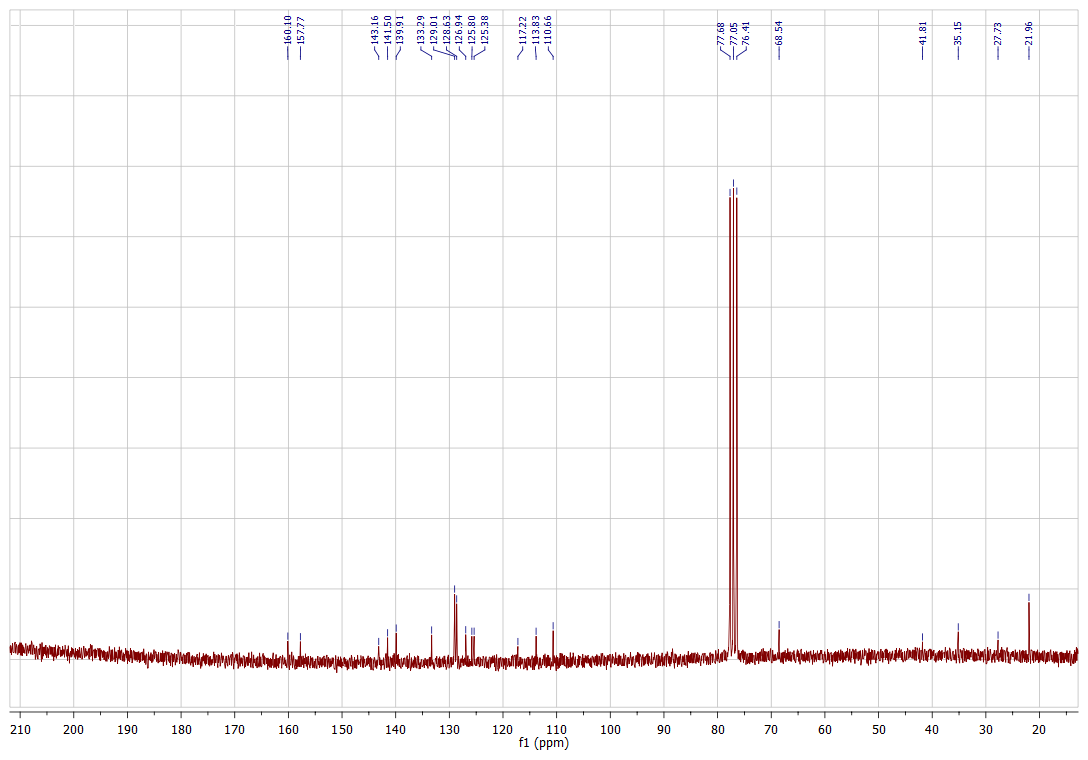
**

**Figure S7.** ^1^H and ^13^C NMR of compound **7g**

**
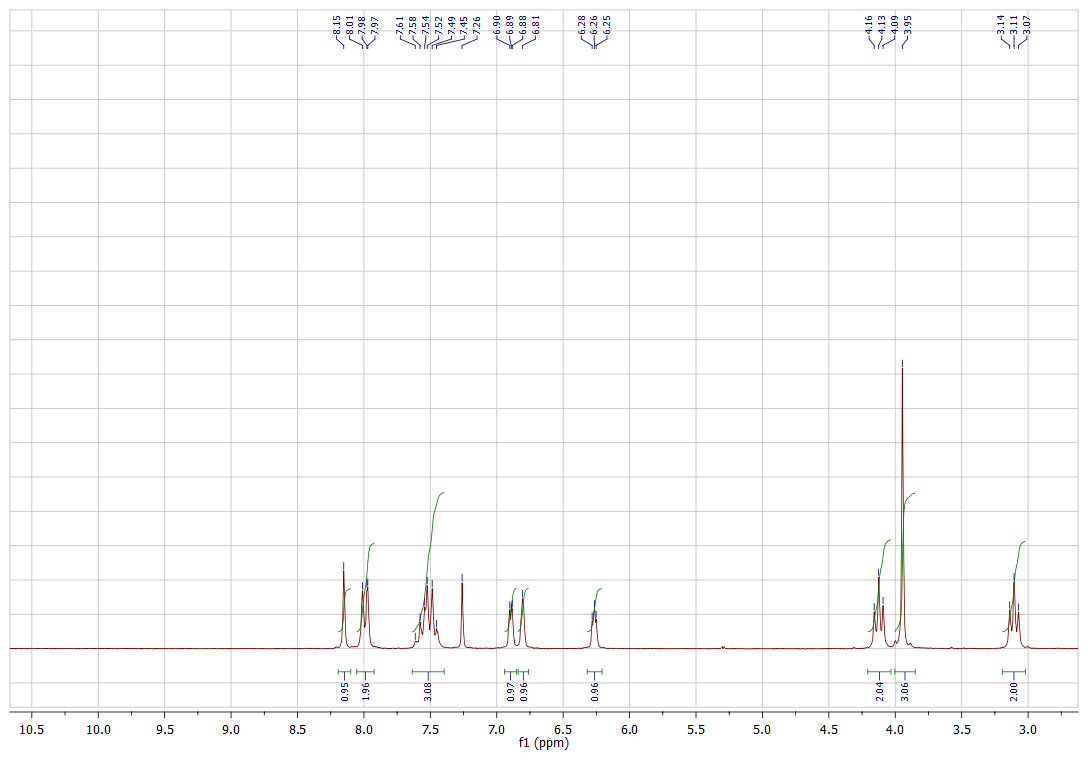
**

**
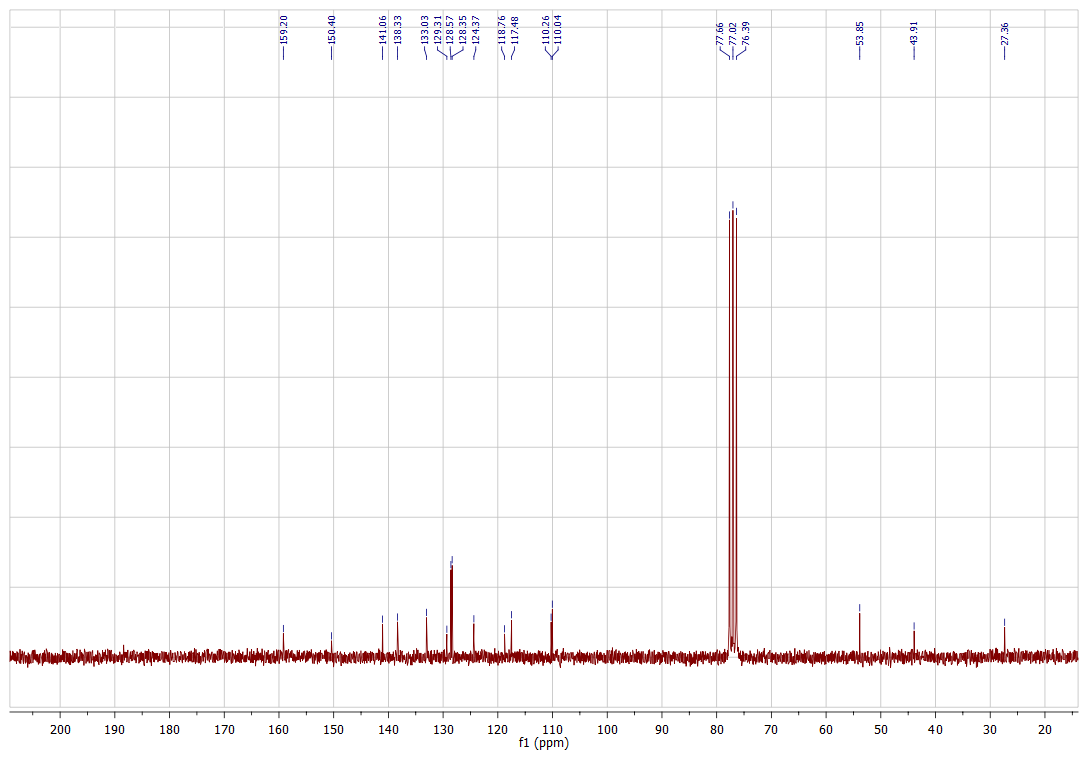
**

**Figure S8.** ^1^H and ^13^C NMR of compound **7h**

**
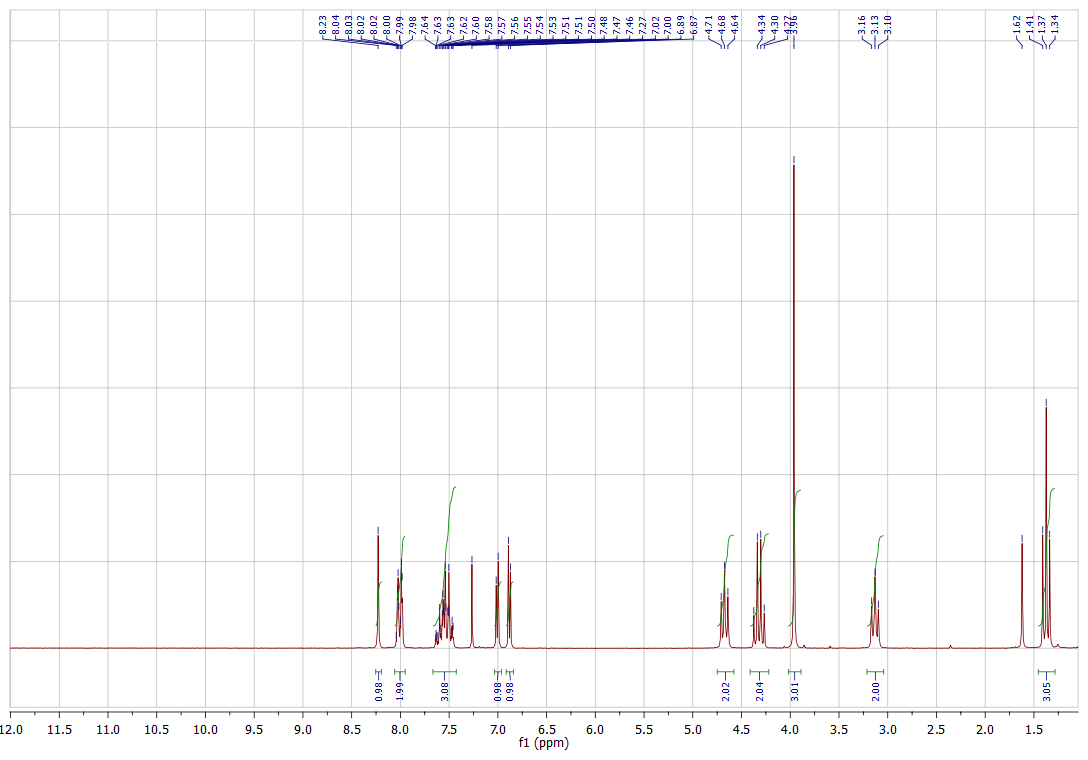
**

**
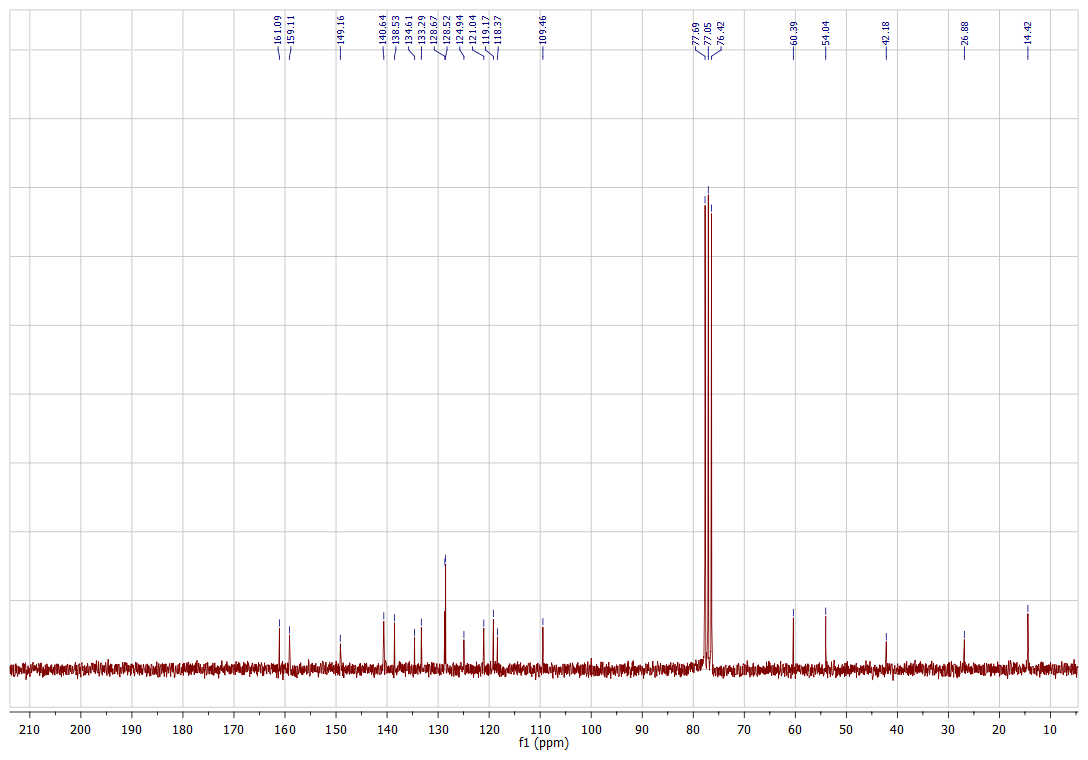
**

**Figure S9.** ^1^H and ^13^C NMR of compound **7i**

**
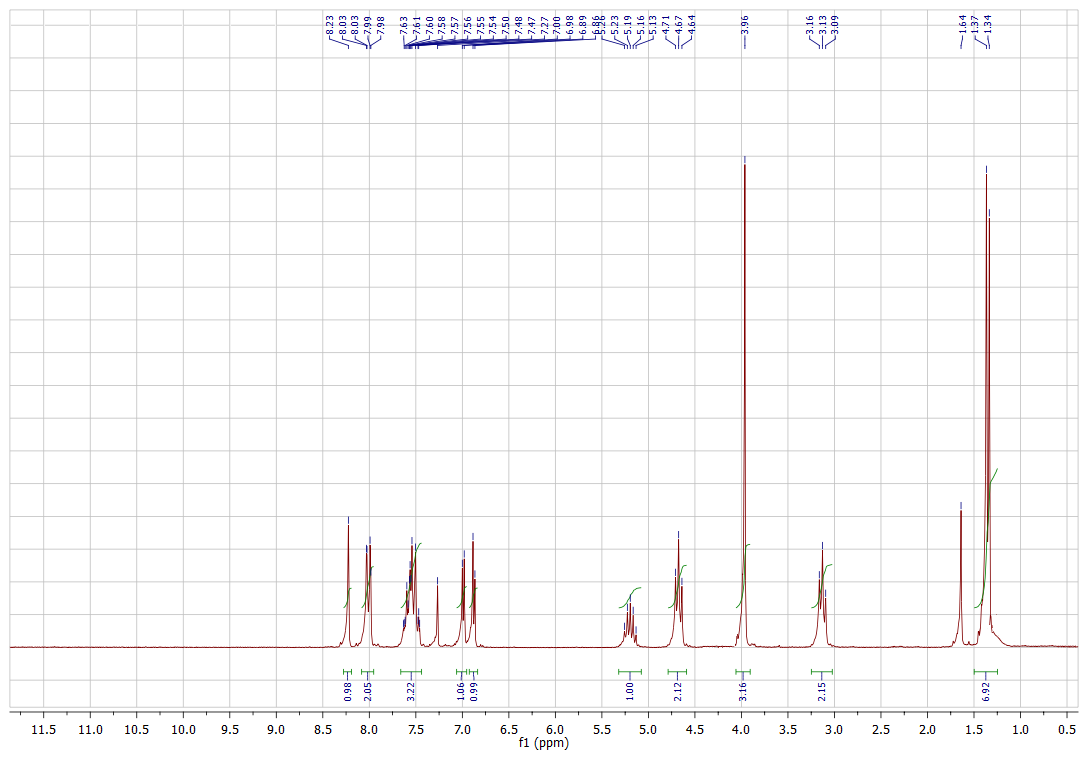
**

**
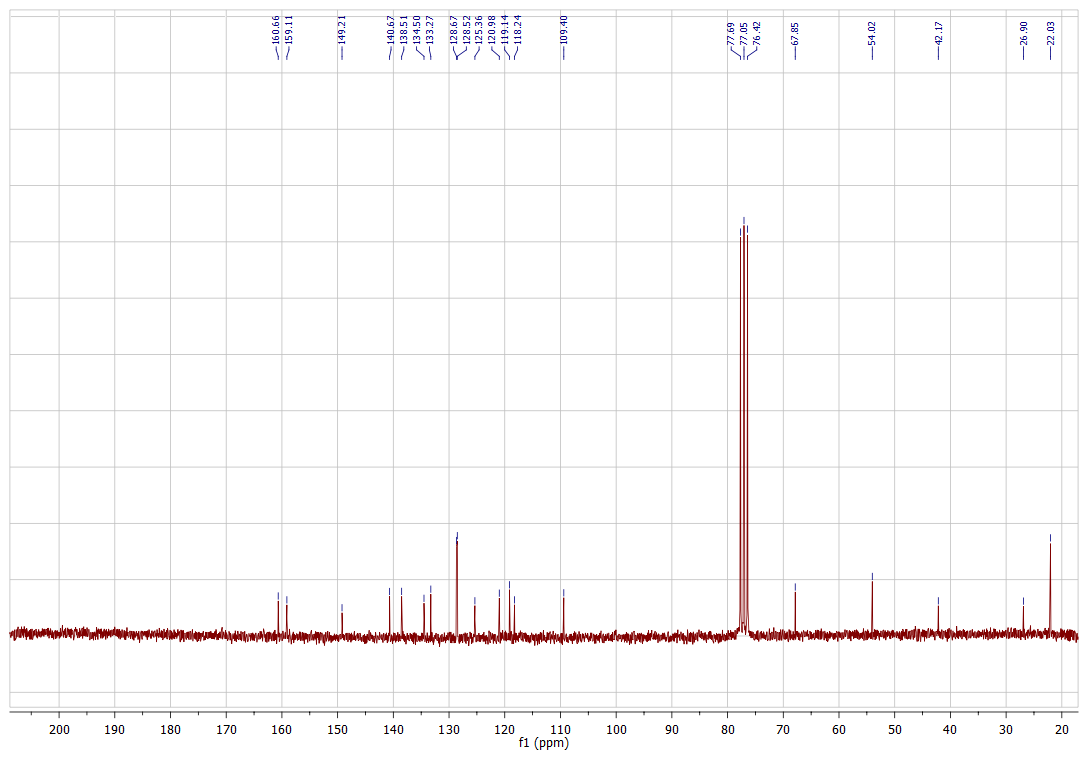
**

**Figure S10.** ^1^H and ^13^C NMR of compound **7j**

**
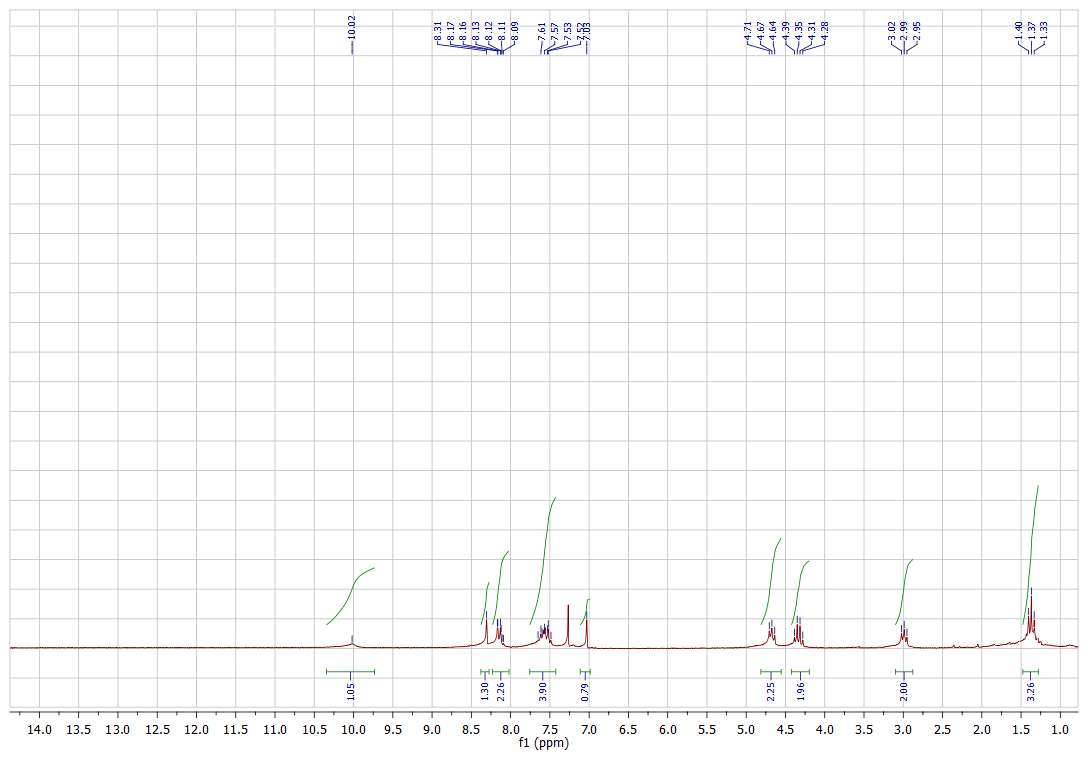
**

**
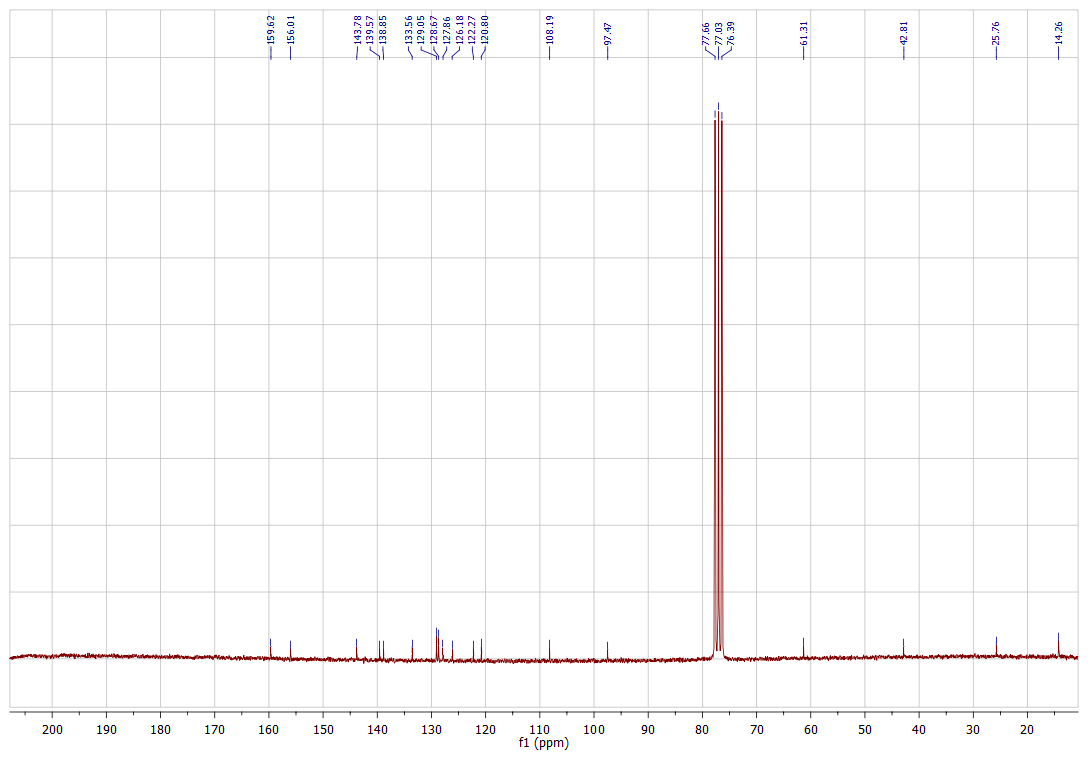
**

**Figure S11.** ^1^H and ^13^C NMR of compound **7k**

**
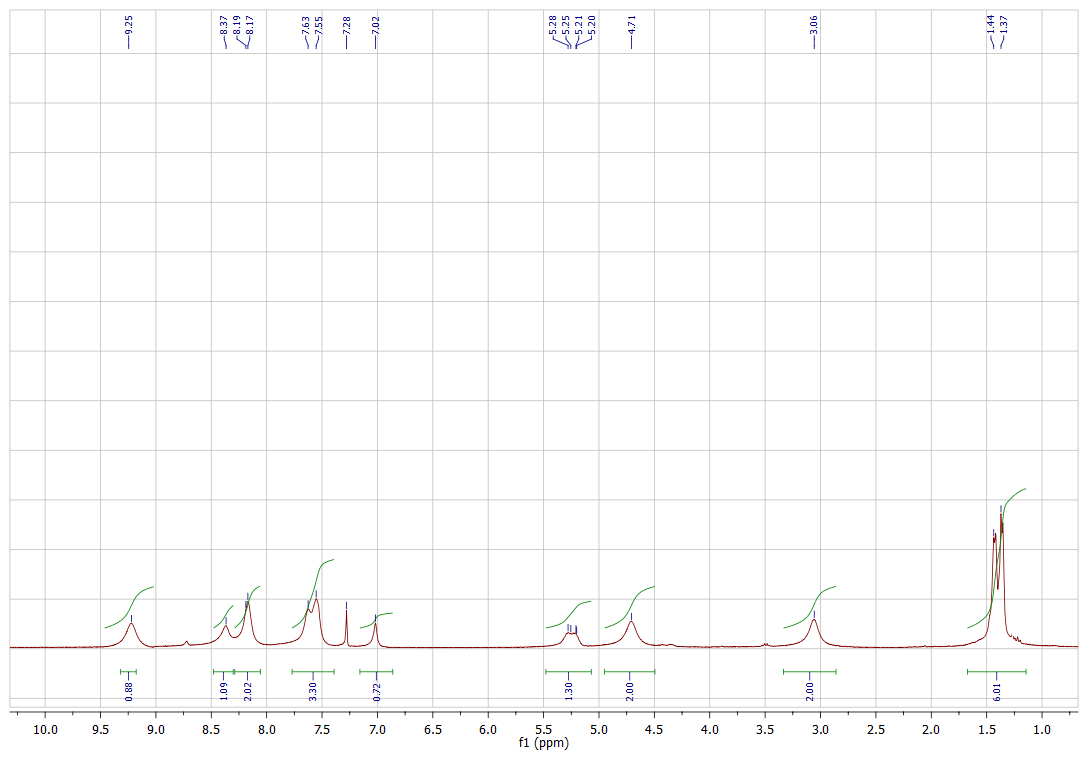
**

**
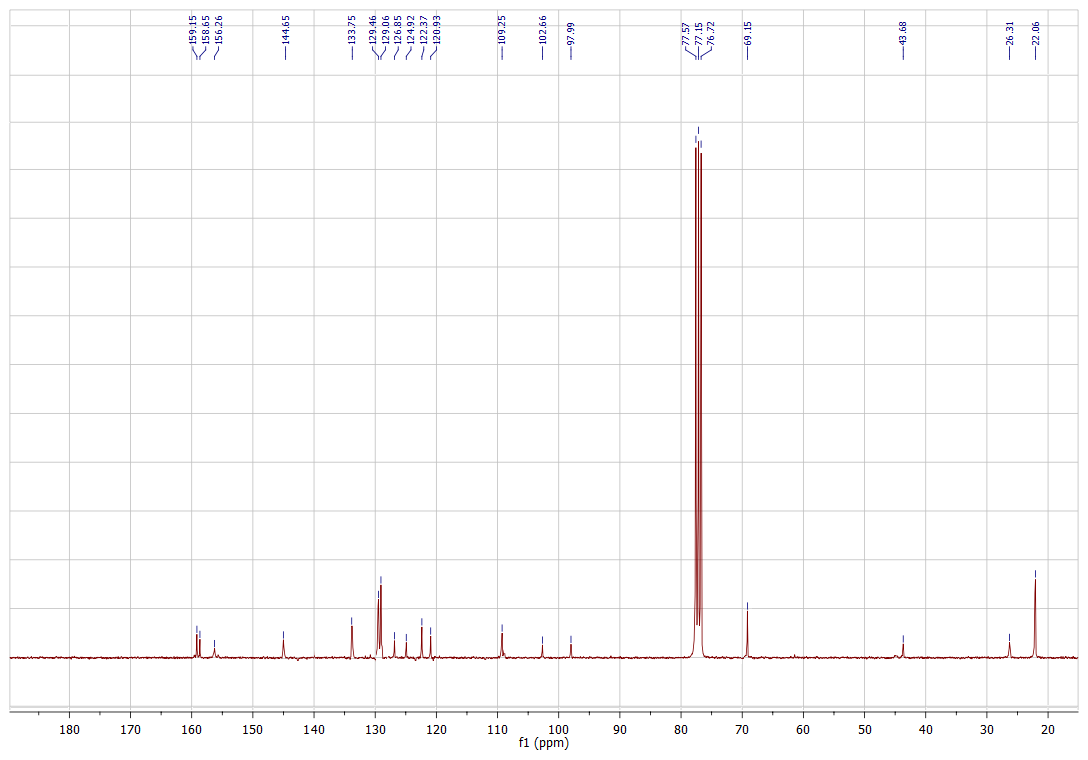
**

**Figure S12.** ^1^H and ^13^C NMR of compound **8a**

**
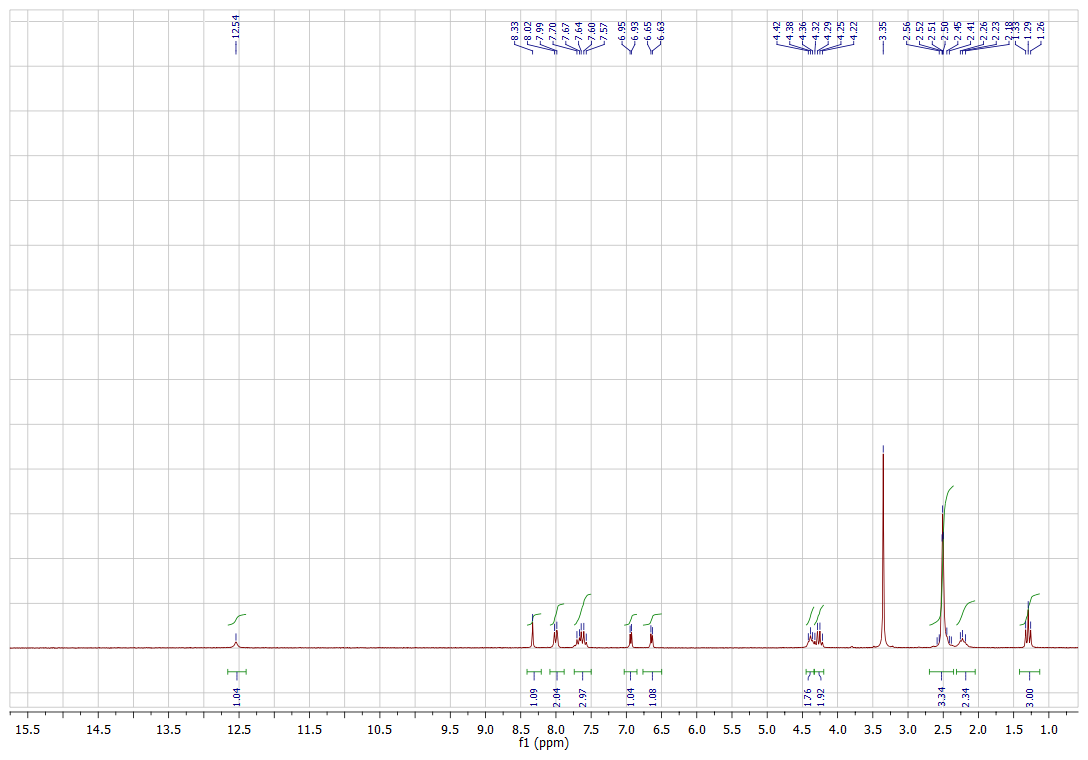
**

**
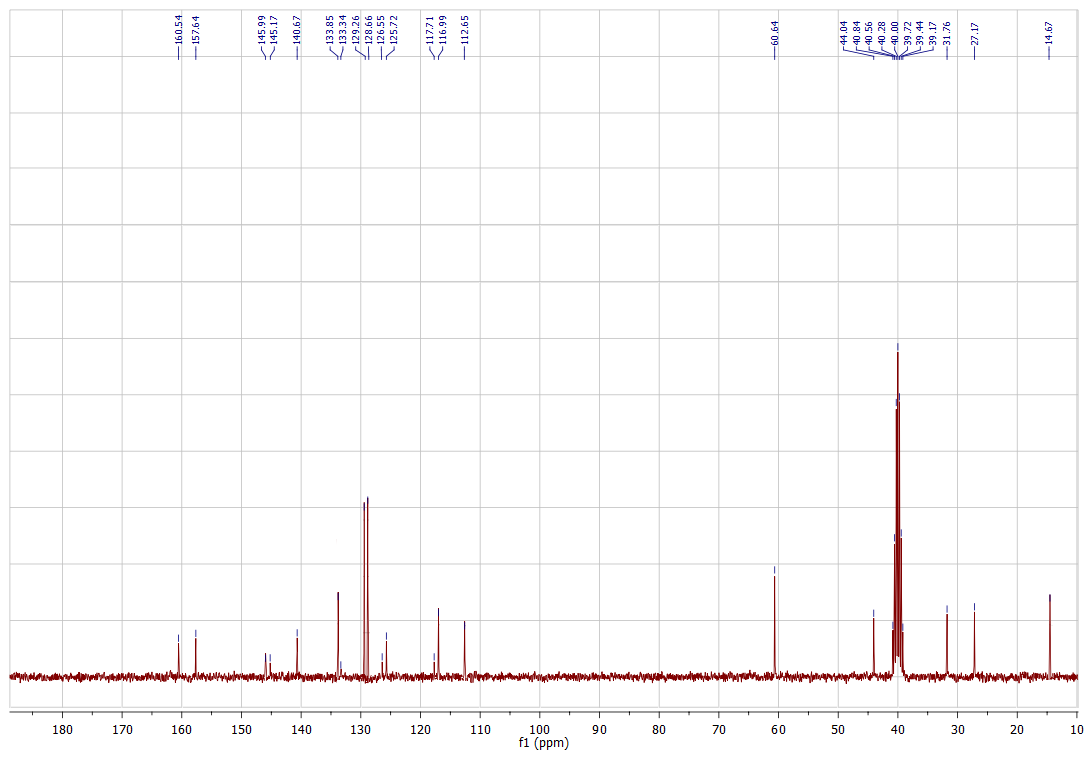
**

**Figure S13.** ^1^H and ^13^C NMR of compound **8b**

**
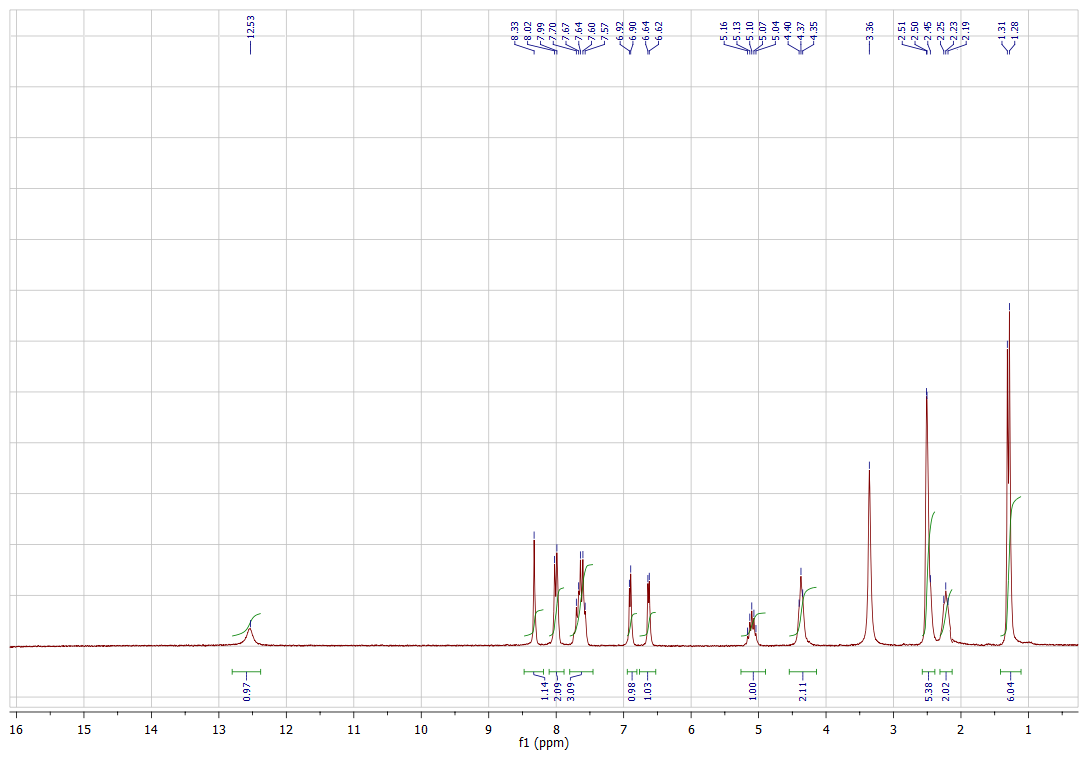
**

**
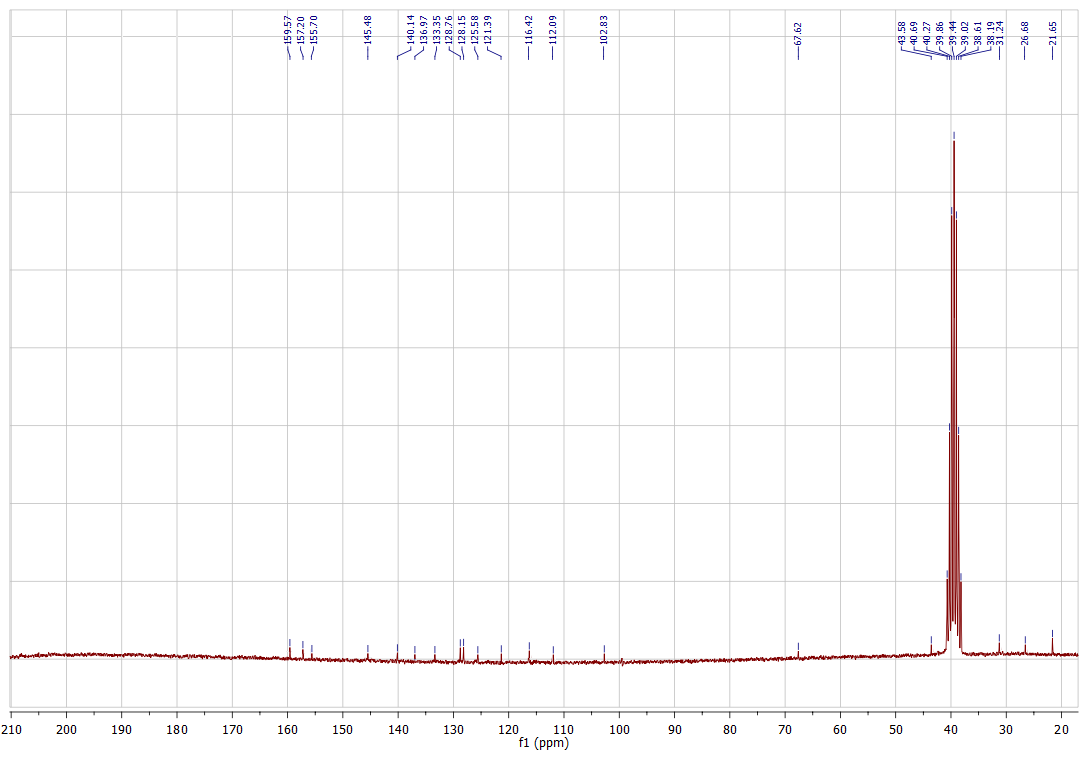
**

**Figure S14.** ^1^H and ^13^C NMR of compound **8c**

**
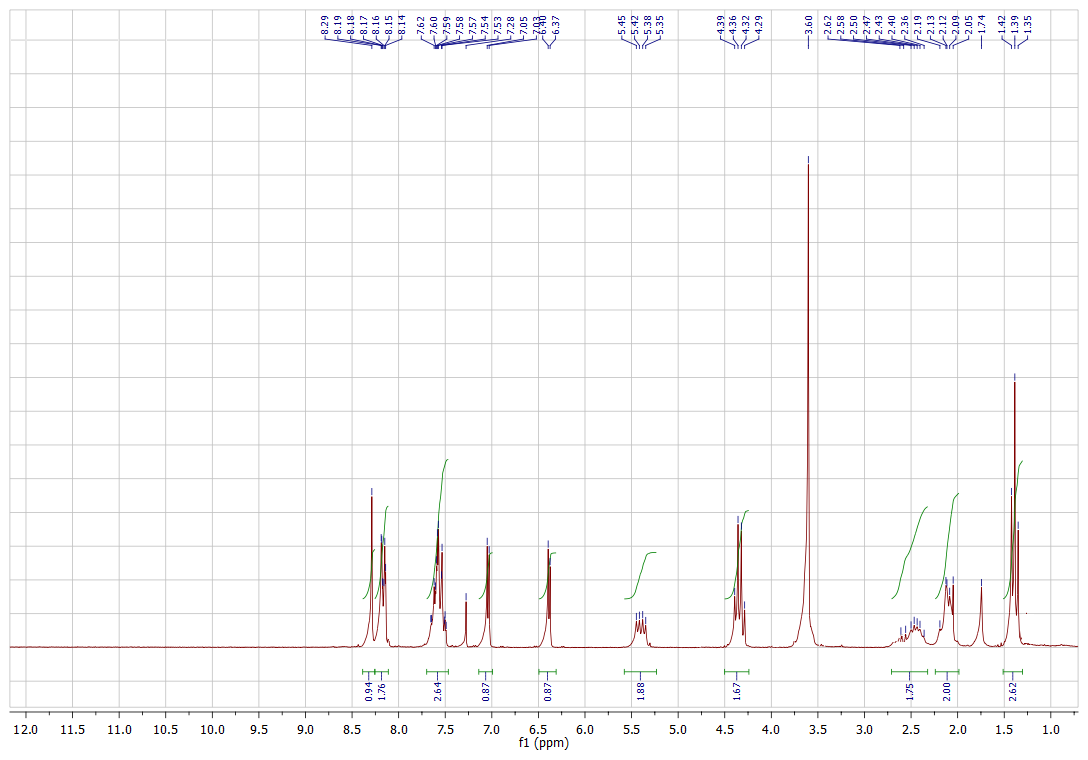
**

**
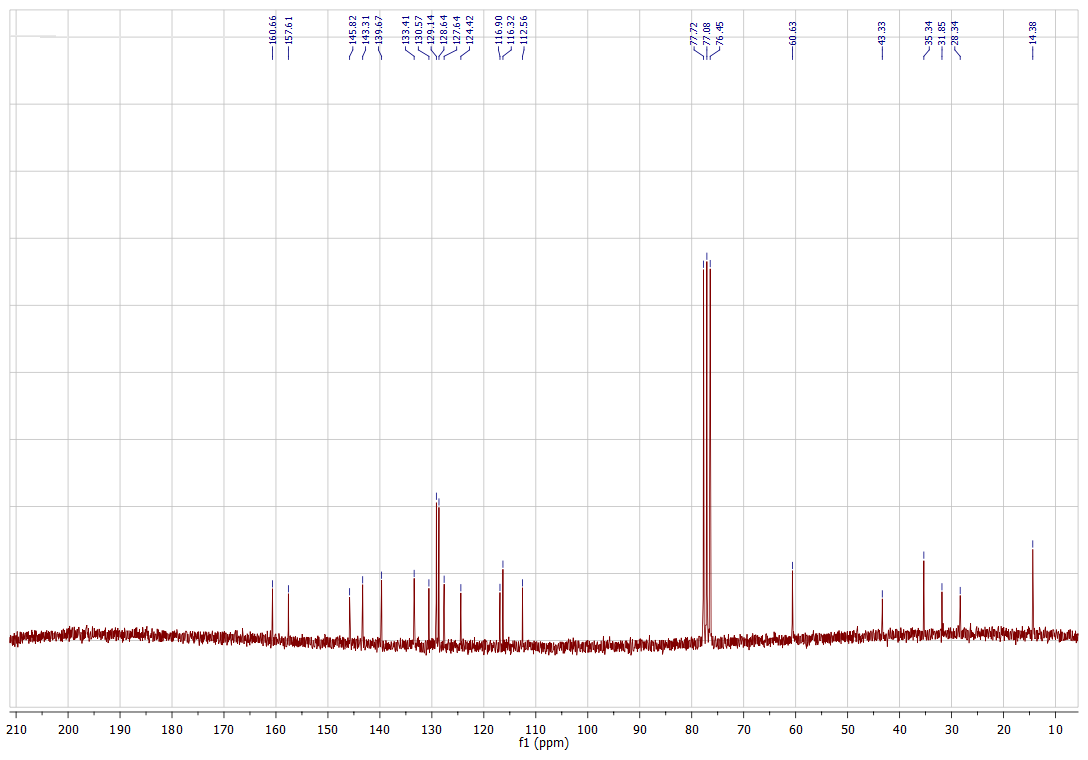
**

**Figure S15.** ^1^H and ^13^C NMR of compound **8d**

**
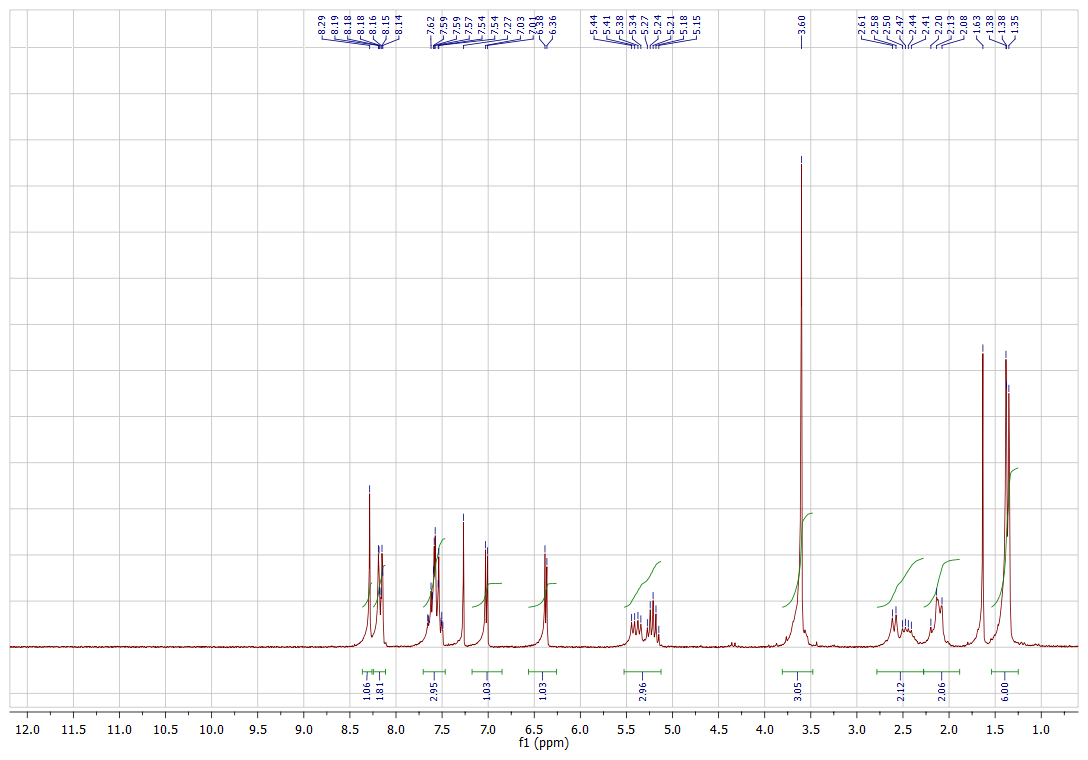
**

**
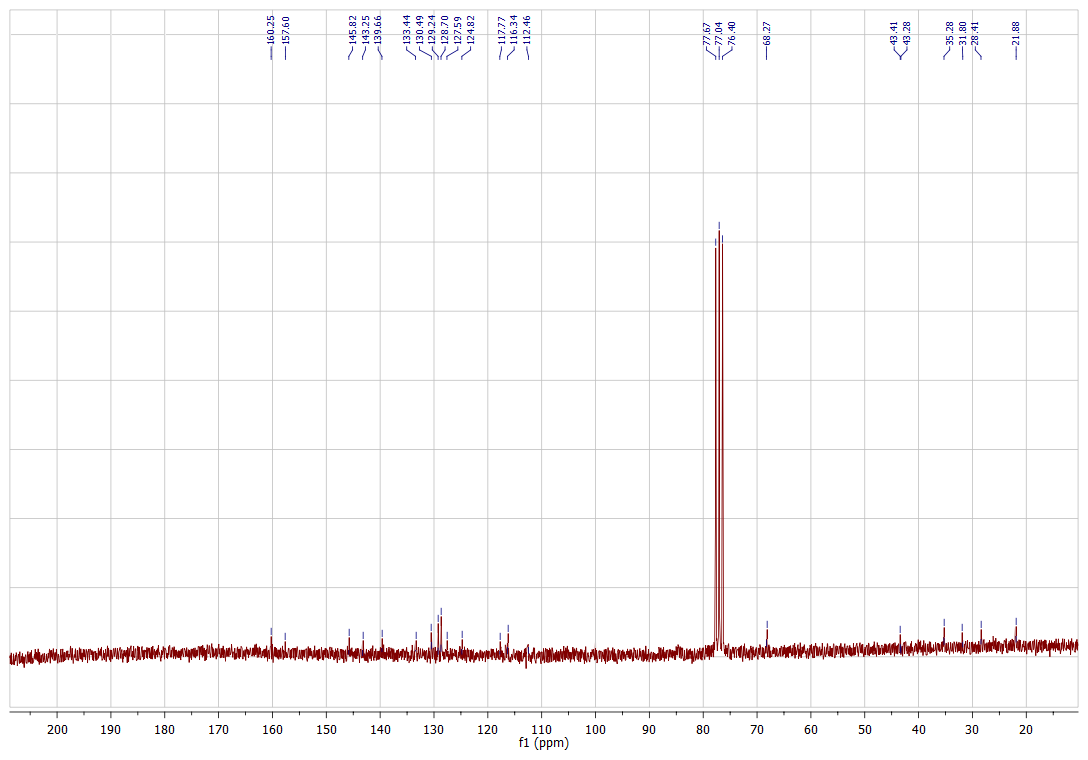
**

**Figure S16.** ^1^H and ^13^C NMR of compound **8e**

**
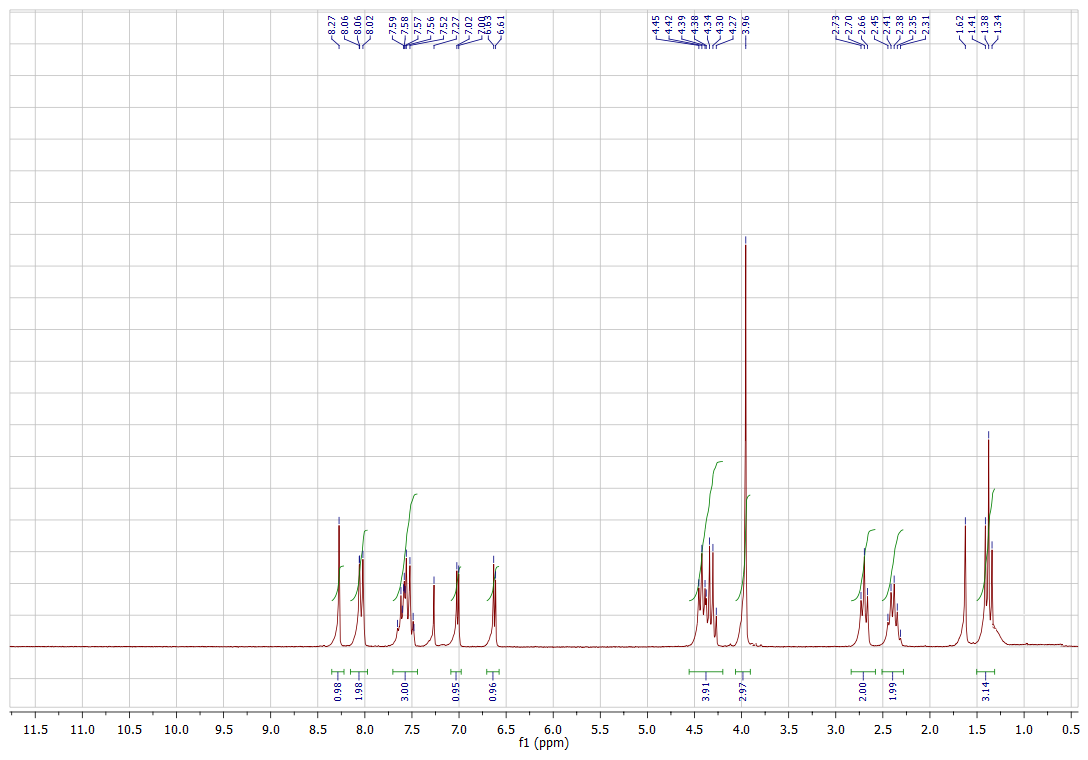
**

**
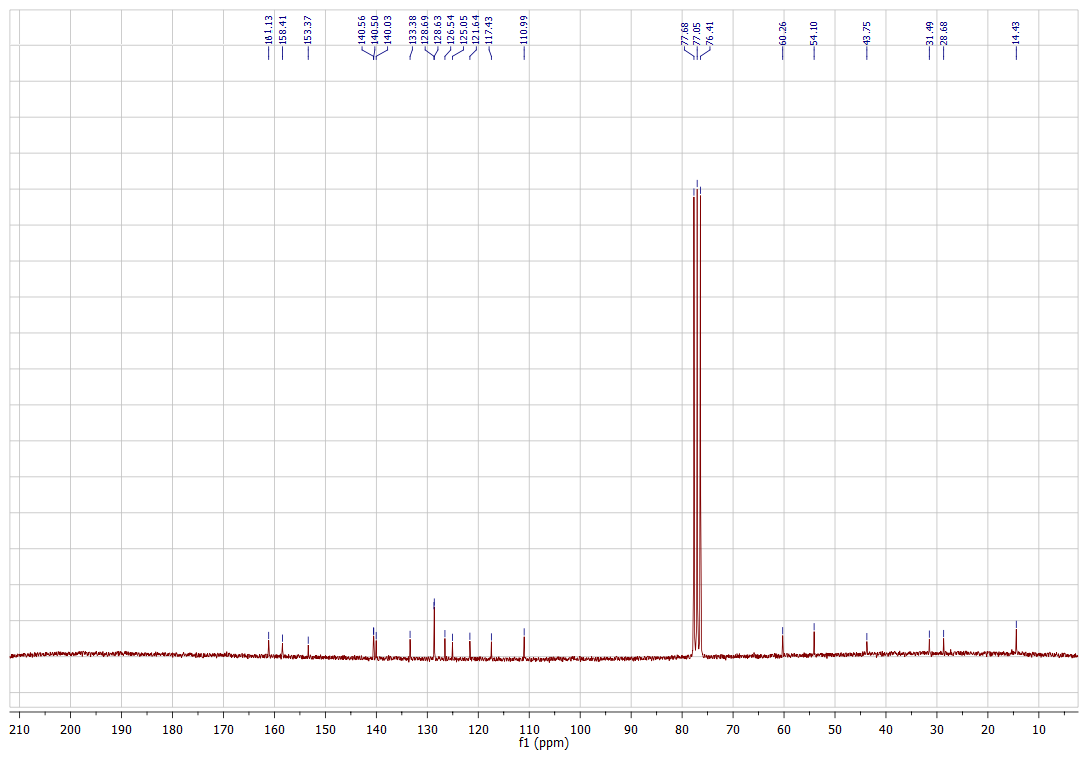
**

**Figure S17.** ^1^H and ^13^C NMR of compound **8f**

**
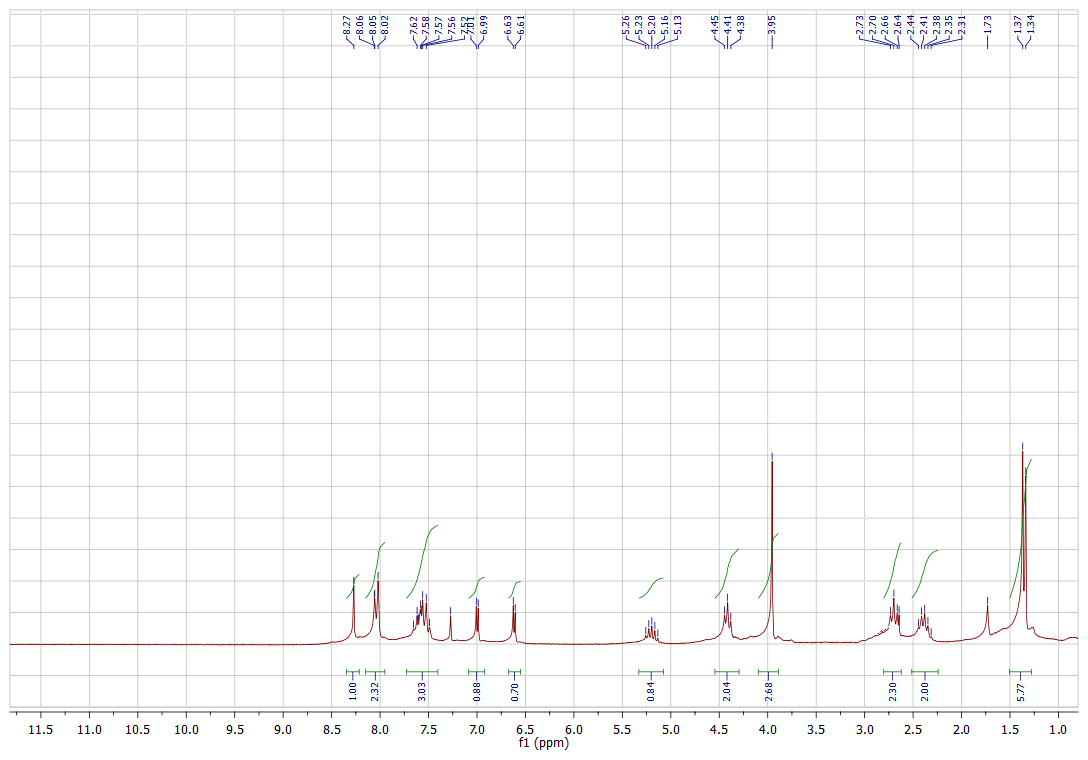
**

**
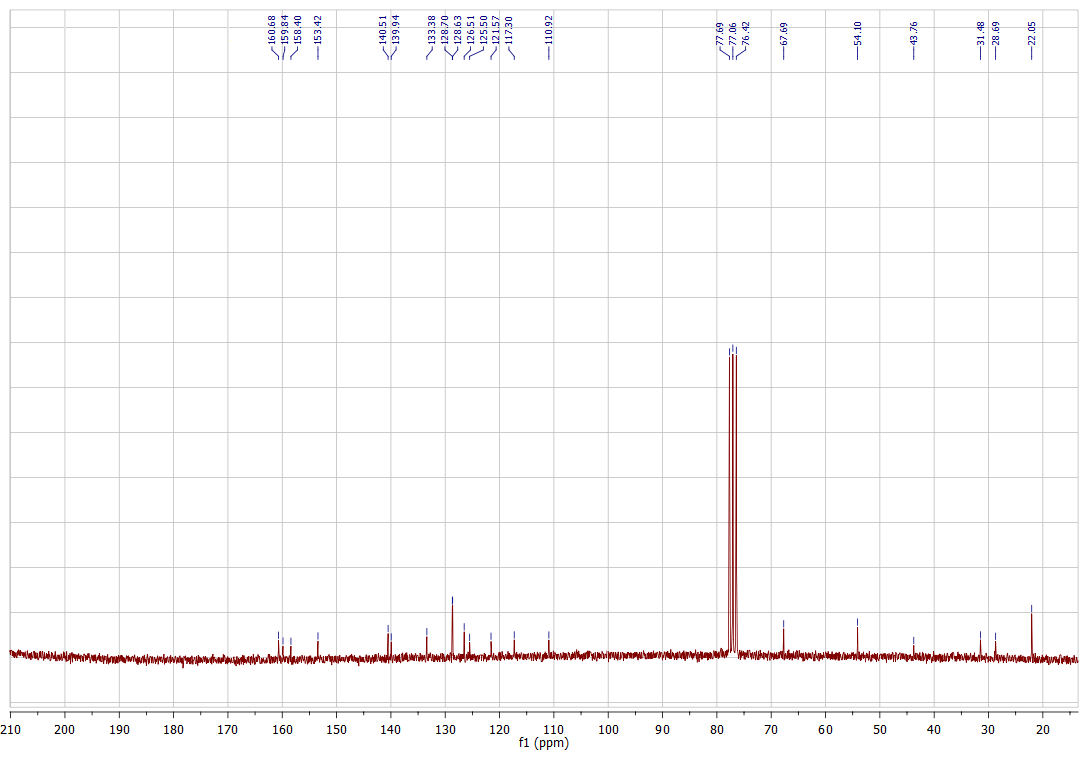
**

**Figure S18.** ^1^H and ^13^C NMR of compound **8g**

**
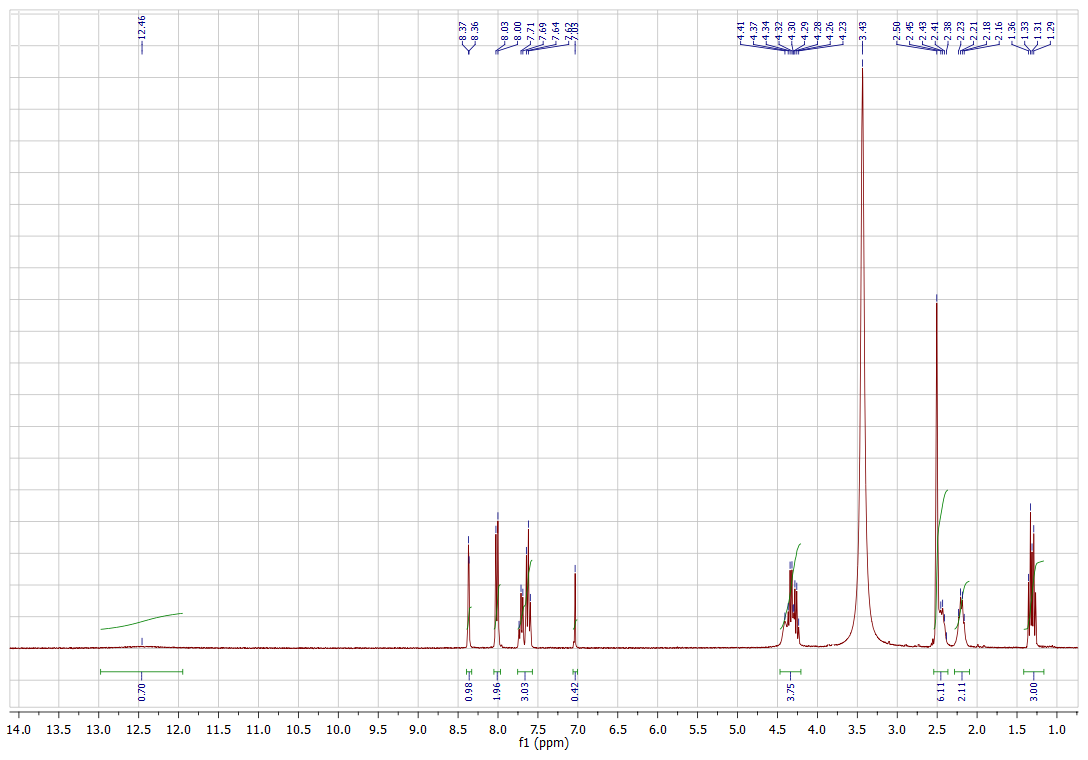
**

**
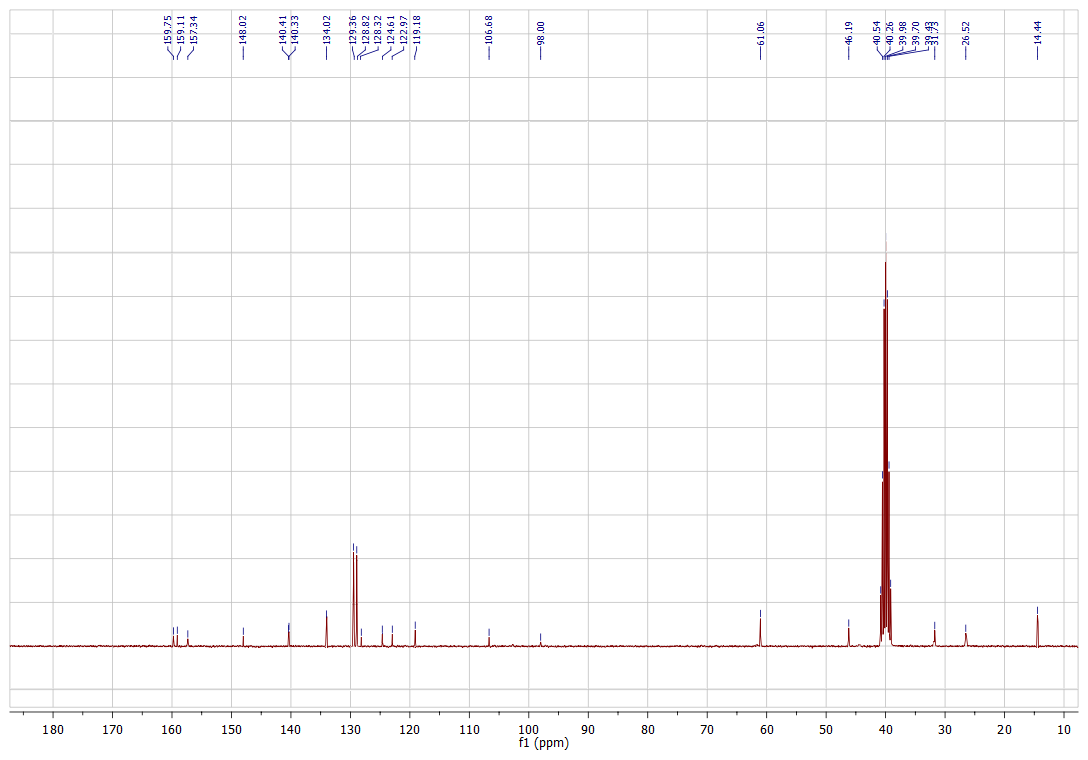
**

**Figure S19.** ^1^H and ^13^C NMR of compound **8h**

**
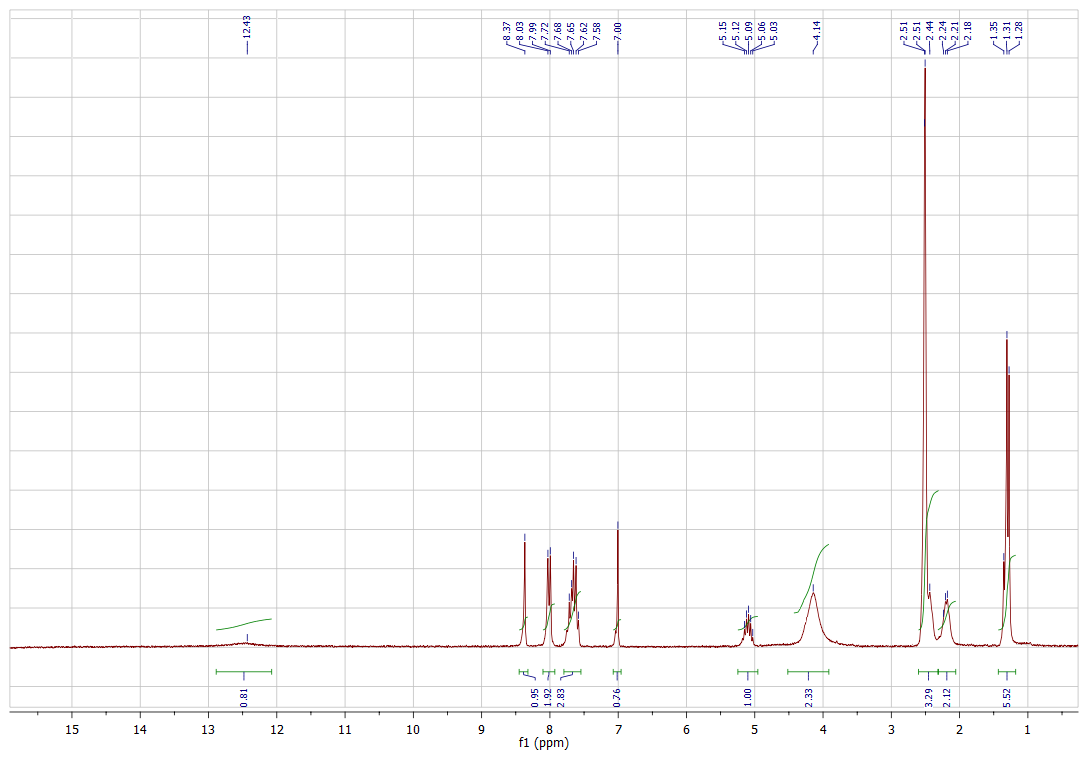
**

**
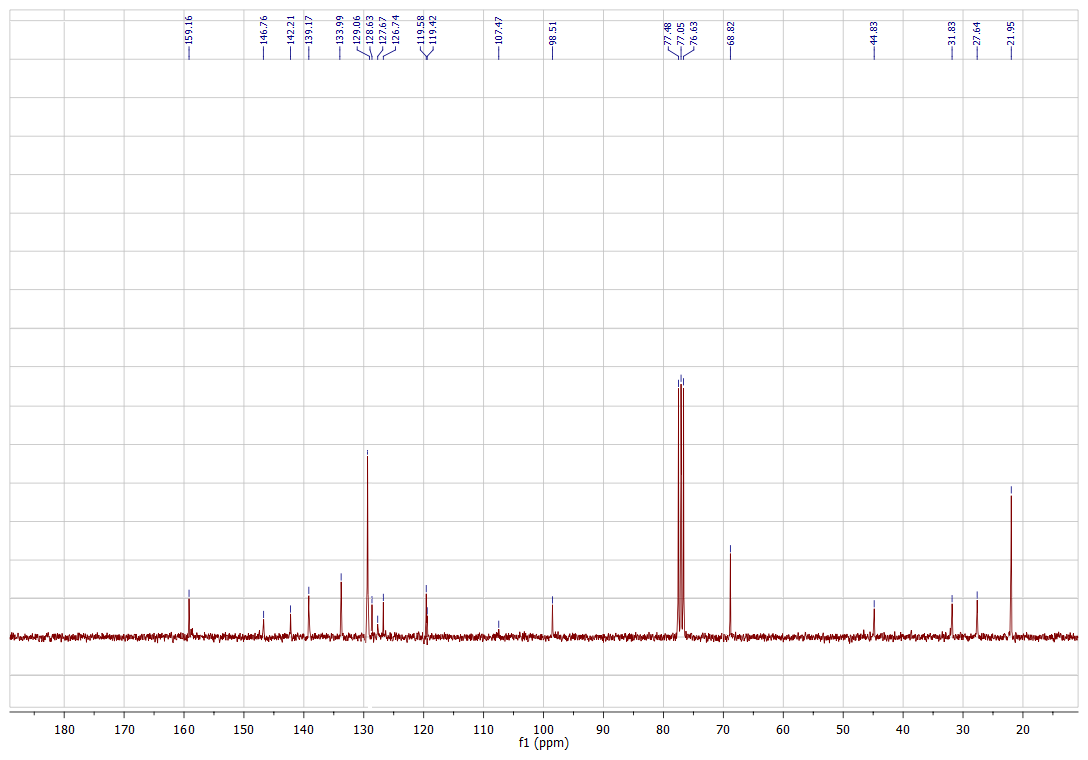
**
